# NUDT7 regulates total hepatic CoA levels and the composition of the intestinal bile acid pool in male mice fed a Western diet

**DOI:** 10.1016/j.jbc.2022.102745

**Published:** 2022-11-24

**Authors:** Schuyler D. Vickers, Stephanie A. Shumar, Dominique C. Saporito, Amina Kunovac, Quincy A. Hathaway, Breeanna Mintmier, Judy A. King, Rachel D. King, Vazhaikkurichi M. Rajendran, Aniello M. Infante, John M. Hollander, Roberta Leonardi

**Affiliations:** 1 Department of Biochemistry and Molecular Medicine, West Virginia University, Morgantown, West Virginia, USA; 2 Division of Exercise Physiology, West Virginia University, Morgantown, West Virginia, USA; 3 Department of Pathology and Translational Pathobiology, LSU Health Shreveport, Shreveport, Louisiana, USA; 4 Genomics Core Facility, West Virginia University, Morgantown, West Virginia, USA

**Keywords:** Nudix hydrolases, peroxisomes, cholesterol, bile acids, dicarboxylic fatty acids, BAAT, bile acid-CoA:amino acid N-acyltransferase, BSH, bile salt hydrolase, CA, cholic acid, CD, control diet, CDCA, chenodeoxycholic acid, CRC, colorectal cancer, DCA, deoxycholic acid, DHCA, dihydroxycholestanoic acid, EP, entrance potential, ER, endoplasmic reticulum, FIT, fat-inducible transcript, HDCA, hyodeoxycholic acid, LCA, lithocholic acid, MCA, muricholic acid, MCD, medium-chain dicarboxylic, TCA, taurocholic acid, TCDCA, taurochenodeoxyholic acid, TDCA, taurodeoxycholic acid, THCA, trihydroxycholestanoic acid, THDCA, taurohyodeoxycholic acid, TLCA, taurolithocholic acid, TMCA, tauromuricholic acid, TUDCA, tauroursodeoxycholic acid, UDCA, ursodeoxycholic acid, WD, Western diet

## Abstract

Nudix hydrolase 7 (NUDT7) is an enzyme that hydrolyzes CoA species, is highly expressed in the liver, and resides in the peroxisomes. Peroxisomes are organelles where the preferential oxidation of dicarboxylic fatty acids occurs and where the hepatic synthesis of the primary bile acids cholic acid and chenodeoxycholic acid is completed. We previously showed that liver-specific overexpression of NUDT7 affects peroxisomal lipid metabolism but does not prevent the increase in total liver CoA levels that occurs during fasting. We generated *Nudt7*^*-/-*^ mice to further characterize the role that peroxisomal (acyl-)CoA degradation plays in the modulation of the size and composition of the acyl-CoA pool and in the regulation of hepatic lipid metabolism. Here, we show that deletion of *Nudt7* alters the composition of the hepatic acyl-CoA pool in mice fed a low-fat diet, but only in males fed a Western diet does the lack of NUDT7 activity increase total liver CoA levels. This effect is driven by the male-specific accumulation of medium-chain dicarboxylic acyl-CoAs, which are produced from the β-oxidation of dicarboxylic fatty acids. We also show that, under conditions of elevated synthesis of chenodeoxycholic acid derivatives, *Nudt7* deletion promotes the production of tauromuricholic acid, decreasing the hydrophobicity index of the intestinal bile acid pool and increasing fecal cholesterol excretion in male mice. These findings reveal that NUDT7-mediated hydrolysis of acyl-CoA pathway intermediates in liver peroxisomes contributes to the regulation of dicarboxylic fatty acid metabolism and the composition of the bile acid pool.

The liver plays a key role in the regulation of whole-body energy metabolism, responding to changes in nutrient availability by taking up circulating glucose and fatty acids for use or storage when they are abundant and by synthesizing and releasing glucose and ketone bodies into the bloodstream during a fast (1, 2). The ability to take up cholesterol from multiple lipoproteins, synthesize this lipid *de novo*, and convert it to cholesteryl esters and bile acids also makes the liver a primary site for the regulation of cholesterol homeostasis (3, 4, 5). Many vital and unique liver functions, including the *de novo* synthesis of glucose, ketone bodies, and bile acids, rely on the availability of CoA. CoA is an essential cofactor that activates a variety of carboxylic acid substrates, thereby allowing them to participate in hundreds of metabolic reactions and in the posttranslational modification of histones and other proteins (6, 7, 8, 9, 10). These processes occur in multiple subcellular compartments, with dedicated pools of CoA and CoA thioesters found in the cytosol, mitochondria, peroxisomes, endoplasmic reticulum (ER), and nucleus (11, 12, 13, 14, 15).

The ability of the liver to switch between fuel sources and maintain whole-body glucose homeostasis in response to fluctuations in the metabolic state is tightly linked to the dynamic regulation of the concentration of total CoA (unacylated CoA plus acyl-CoAs) (16, 17, 18). Activation of the hepatic CoA biosynthetic pathway drives the increase in total CoA that is observed in this organ in the fasted state (17, 19). Conversely, the decrease in total CoA levels that occurs upon refeeding requires degradation of the CoA and acyl-CoAs accumulated during the fast. Degradation of CoA species to 3′,5′-ADP and (acyl-)phosphopantetheine is catalyzed by three specific Nudix hydrolases, NUDT7, NUDT8, and NUDT19 (20). NUDT7 and NUDT19 are both peroxisomal enzymes, whose expression is almost completely restricted to the liver and kidneys, respectively (21, 22, 23, 24). Hepatic NUDT7 levels respond to changes in the nutritional state and are highest in the fed state, when the total CoA content of the liver decreases (18, 24). NUDT8 localizes to the mitochondria, has a unique dependence on Mn^2+^ for activity, and exhibits a broader tissue distribution in the liver, kidneys, heart, and brown adipose tissue, compared to NUDT7 and NUDT19 (25). In addition to these Nudix hydrolases, the broadly expressed, ER-resident, fat-inducible transcript (FIT) protein, FIT2, has been recently shown to possess acyl-CoA diphosphohydrolase activity towards unsaturated long-chain acyl-CoAs (26). The existence of (acyl-)CoA-degrading enzymes in multiple subcellular compartments suggests that these enzymes could work to locally modulate the size and composition of subcellular (acyl-)CoA pools. This function could not only contribute to the regulation of total CoA levels but also control the specific (acyl-)CoA-dependent processes that occur in each subcellular compartment.

Peroxisomes are essential organelles for lipid metabolism. They are indispensable for the synthesis of plasmalogens and are the exclusive site for the oxidation of branched-chain and very long-chain fatty acids (27, 28, 29). The oxidation of medium-chain dicarboxylic (MCD) and long-chain dicarboxylic fatty acids, which is stimulated under conditions that increase fatty acid supply, including fasting and diabetes (30, 31), takes place preferentially in the peroxisomes (32, 33, 34). These organelles are also where the last, CoA-dependent steps in the bile acid biosynthetic pathway occur (35). Bile acids are amphipathic compounds that aid in the intestinal absorption of dietary lipids and act as signaling molecules; they bind to multiple receptors to regulate their own biosynthesis and a variety of other processes including glucose and lipid metabolism (36, 37, 38, 39). The major pathway for the synthesis of bile acids, the classic pathway, starts with the hydroxylation of cholesterol to 7α-hydroxycholesterol, catalyzed by the first and rate-limiting enzyme, cholesterol 7α-hydroxylase (CYP7A1) (40, 41). A major branch of this pathway then proceeds to the formation of 3α,7α-dihydroxycholestanoic acid (DHCA), the precursor of chenodeoxycholic acid (CDCA). The other branch of the pathway generates 3α,7α,12α-trihydroxycholestanoic acid (THCA), the precursor of cholic acid (CA). The production of THCA requires sterol 12-alpha-hydroxylase (CYP8B1), an enzyme that adds a hydroxyl group to carbon 12 of the steroid core (42, 43). This activity controls the ratio between CDCA and CA and the hydrophilic-hydrophobic balance of the bile acid pool, which, in turn, affects lipid absorption (44, 45). The alternative pathway for bile acid synthesis is initiated by the hydroxylation of the side chain of cholesterol by sterol 27-hydroxylase (CYP27A1), followed by 7α-hydroxylation by oxysterol 7α-hydroxylase (CYP7B1), and predominantly produces DHCA (46, 47). Following activation of DHCA and THCA to their respective CoA thioesters, these bile acid precursors enter the peroxisomes where they undergo side chain β-oxidation to yield CA-CoA and CDCA-CoA, respectively. These acyl-CoAs can then be hydrolyzed to CA and CDCA or used as substrates by the enzyme bile acid-CoA:amino acid N-acyltransferase (BAAT). BAAT allows for the formation of bile acids conjugated to glycine or taurine, with mice forming predominantly taurinated bile acids (48). Within liver peroxisomes, the CoA-bound bile acid precursors could also be degraded by NUDT7. Indeed, recombinant NUDT7 can readily hydrolyze a variety of CoA species, including THCA-CoA and CA-CoA (23, 24) and, when overexpressed in the liver, NUDT7 decreases the hepatic concentration of CA-CoA and both CA- and CDCA-derived primary and secondary bile acids (49). In fasted mice, exogenously expressed NUDT7 also diminishes the rate of peroxisomal fatty acid oxidation, suggesting a role for this enzyme in the regulation of peroxisomal lipid metabolism in the liver. Interestingly, increasing peroxisomal (acyl-)CoA degradation is not sufficient to prevent the fasting-induced elevation of total hepatic CoA levels, and it is presently unknown whether NUDT7 activity contributes to the net decrease in total CoA that occurs in the liver of fed mice.

*Nudt7*^*-/-*^ mice have been recently generated (50, 51). These mice exhibit compromised cartilage integrity and the propensity to develop more colonic polyps, but the effect of *Nudt7* deletion on (acyl-)CoA levels and global hepatic metabolism has yet to be characterized. Here, we show that, depending on the diet, NUDT7 activity regulates not only the size and composition of the hepatic acyl-CoA pool, but also the hydrophobicity index of the intestinal bile acid pool and fecal cholesterol excretion, in a gender-specific manner.

## Results

### NUDT7 contributes to the regulation of the acyl-CoA pool composition, but not total CoA levels, in the liver of mice fed a low-fat diet

**Figure 1:**
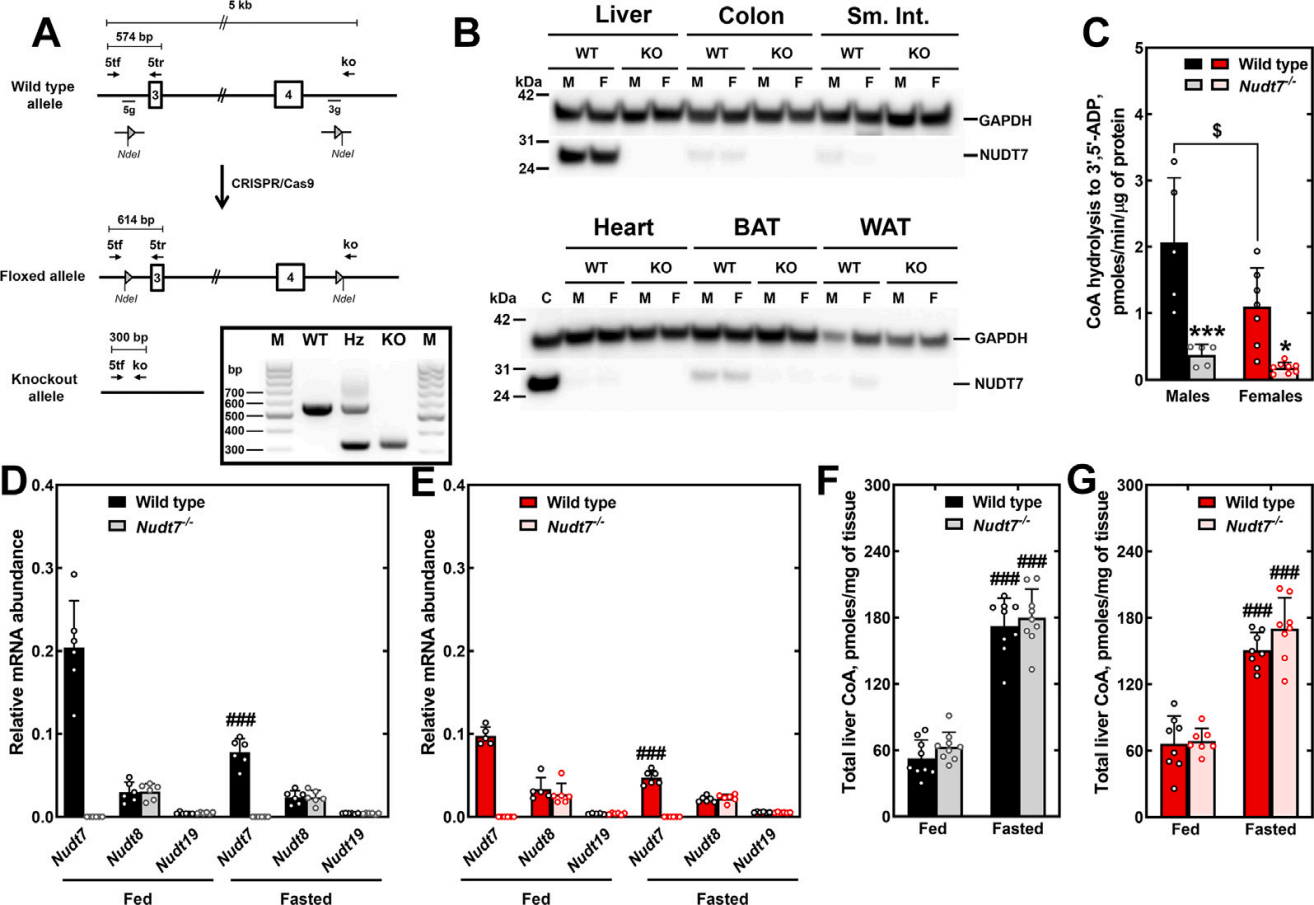
**Deletion of *Nudt7* reduces theliverCoA-degrading activitywithout affecting total CoA levels in mice fed the CD**. *A*, strategy for the generation of the *Nudt7*^*-/-*^ mice (see Experimental procedures). Rectangles represent exons 3 and 4, as numbered, and triangles represent LoxP sites. The position of primers used for genotyping (inset) is shown. M, marker; Hz, heterozygous. *B*, NUDT7 tissue distribution and confirmation of successful *Nudt7* deletion. M, male; F, female; Sm. Int., small intestine; BAT, brown adipose tissue; WAT, white adipose tissue. Liver homogenate from a WT mouse was used as a positive control, C. *C*, total CoA-degrading activity in liver homogenates. *D* and *E*, *Nudt7*, *Nudt8*, and *Nudt19* transcript levels in the livers of male (*D*) and female (*E*) mice in the fed and 24 h–fasted states. *F* and *G*, total liver CoA levels in fed and 24 h–fasted male (*F*) and female (*G*) mice. Data are shown as the mean (*bar*) of measurements conducted on individual mice (*circles*) ± SD. Two-way ANOVA, $ *p* < 0.05 comparing males to females of the same genotype; ∗*p* < 0.05, ∗∗∗*p* < 0.001 comparing WT to KO mice of the same gender; ### *p* < 0.001 between mice of the same genotype in the fed *versus* fasted state. CD, control diet.

We generated mice with a global deletion of the *Nudt7* gene using CRISPR/Cas9 genome editing (Fig. 1*A*). *Nudt7*^*-/-*^ mice showed complete loss of NUDT7 in the liver, where this protein is predominantly expressed, and also in the colon, small intestine, and brown adipose tissue, where NUDT7 levels are low but still detectable (Fig. 1*B*). Lack of hepatic NUDT7 led to a ∼80% decrease in the total CoA diphosphohydrolase activity detected in liver homogenates incubated with CoA and Mn^2+^, conditions that also support the activity of NUDT19 and NUDT8 (22, 25) (Fig. 1*C*). The dramatic decrease in the generation of the hydrolytic product 3′,5’-ADP in liver homogenates from the *Nudt7*^*-/-*^ mice supports the conclusion that NUDT7 is a major CoA-degrading enzyme in the liver. No compensatory increase in the hepatic expression of *Nudt8*, *Nudt19*, and *Fit2* was observed in response to *Nudt7* deletion in mice of either gender (Figs. 1, *D*, *E*, S2, *E*, and *H*). The enzymatic assays and the gene expression analysis also suggested that hepatic NUDT7 levels might be lower in WT females than WT males, which was confirmed by Western blot analysis (Fig. S2, *A*–*D*). This difference was specific to NUDT7, as the concentration of catalase, a peroxisomal marker, was similar between genders. Male and female *Nudt7*^*-/-*^ mice fed a low-fat diet (control diet, CD) exhibited similar body weight, blood glucose, serum, and liver lipids, compared to control mice (Table 1). Additionally, deletion of *Nudt7* had no effect on total CoA levels in either males or females (Fig. 1, *F* and *G*), indicating that the decrease in the concentration of total CoA observed in the liver of *ad libitum* fed mice, compared to mice fasted for 24 h, was not driven by NUDT7.

**Table 1:** Selected features of fed and 24h-fasted *Nudt7*^*-/*-^ (KO) and WT mice

Parameter	Genotype	Males		Females	
		CD	WD	CD	WD
Body weight, 16 weeks, g	WT	29.1 ± 2.9	32.0 ± 4.2^#^	21.4 ± 1.1	22.8 ± 1.9
	KO	27.1 ± 1.5	31.8 ± 1.5^##^	20.9 ± 2.4	22.7 ± 2.1
Blood glucose, mg/dl	WT	93 ± 14	104 ± 24	112 ± 12	99 ± 14
	KO	101 ± 12	104 ± 15	99 ± 18	92 ± 21
Blood glucose, fasted, mg/dl	WT	62 ± 11	68 ± 11	53 ± 7	52 ± 8
	KO	64 ± 10	67 ± 12	60 ± 13	51 ± 10
Serum Alanine Transaminase, U/l	WT	20 ± 11	25 ± 10	21 ± 6	28 ± 18
	KO	48 ± 35	38 ± 16	28 ± 7	18 ± 5
Serum TAG, mg/dl	WT	79 ± 30	50 ± 26^#^	49 ± 31	63 ± 18
	KO	55 ± 30	47 ± 22	59 ± 27	49 ± 12
Serum TAG, fasted, mg/dl	WT	54 ± 17	62 ± 9	38 ± 8	58 ± 10^#^
	KO	48 ± 19	59 ± 5	36 ± 10	50 ± 16
Serum cholesterol, mg/dl	WT	103 ± 29	170 ± 26^###^	78 ± 25	143 ± 32^###^
	KO	89 ± 35	157 ± 36^###^	87 ± 22	132 ± 15^###^
Serum cholesterol, fasted, mg/dl	WT	115 ± 23	139 ± 56	113 ± 26	123 ± 10
	KO	106 ± 12	144 ± 46	120 ± 64	113 ± 17
Serum C4, nM	WT	36 ± 24	120 ± 24^##^	69 ± 36	267 ± 117^###^
	KO	36 ± 17	122 ± 80^##^	58 ± 43	187 ± 38^##^
Liver weight, mg/g body weight	WT	41.6 ± 3.8	42.4 ± 2.4	44.5 ± 4.0	52.0 ± 4.0^##^
	KO	40.7 ± 1.3	45.7 ± 6.0	44.8 ± 4.4	48.2 ± 2.6
Liver TAG, mg/g of liver	WT	14.5 ± 10.0	79.2 ± 40.1^##^	20.6 ± 11.4	58.7 ± 31.5^###^
	KO	18.8 ± 3.9	80.5 ± 81.2^##^	18.7 ± 11.6	52.8 ± 29.2^##^
Liver cholesterol, mg/g liver	WT	0.65 ± 0.21	2.01 ± 1.35^#^	1.65 ± 0.28	3.86 ± 0.73^###^
	KO	0.69 ± 0.24	1.72 ± 1.57	1.16 ± 0.29	4.57 ± 0.75^###^

Values are expressed as the mean of 6 to 23 mice per condition ± SD. Two-way ANOVA, # *p* ≤ 0.05, ## *p* ≤ 0.01, and ### *p* ≤ 0.001 between mice of the same genotype-fed different diets. TAG, triacylglycerol, C4, 7α-hydroxy-4-cholesten-3-one.

**Figure 2:**
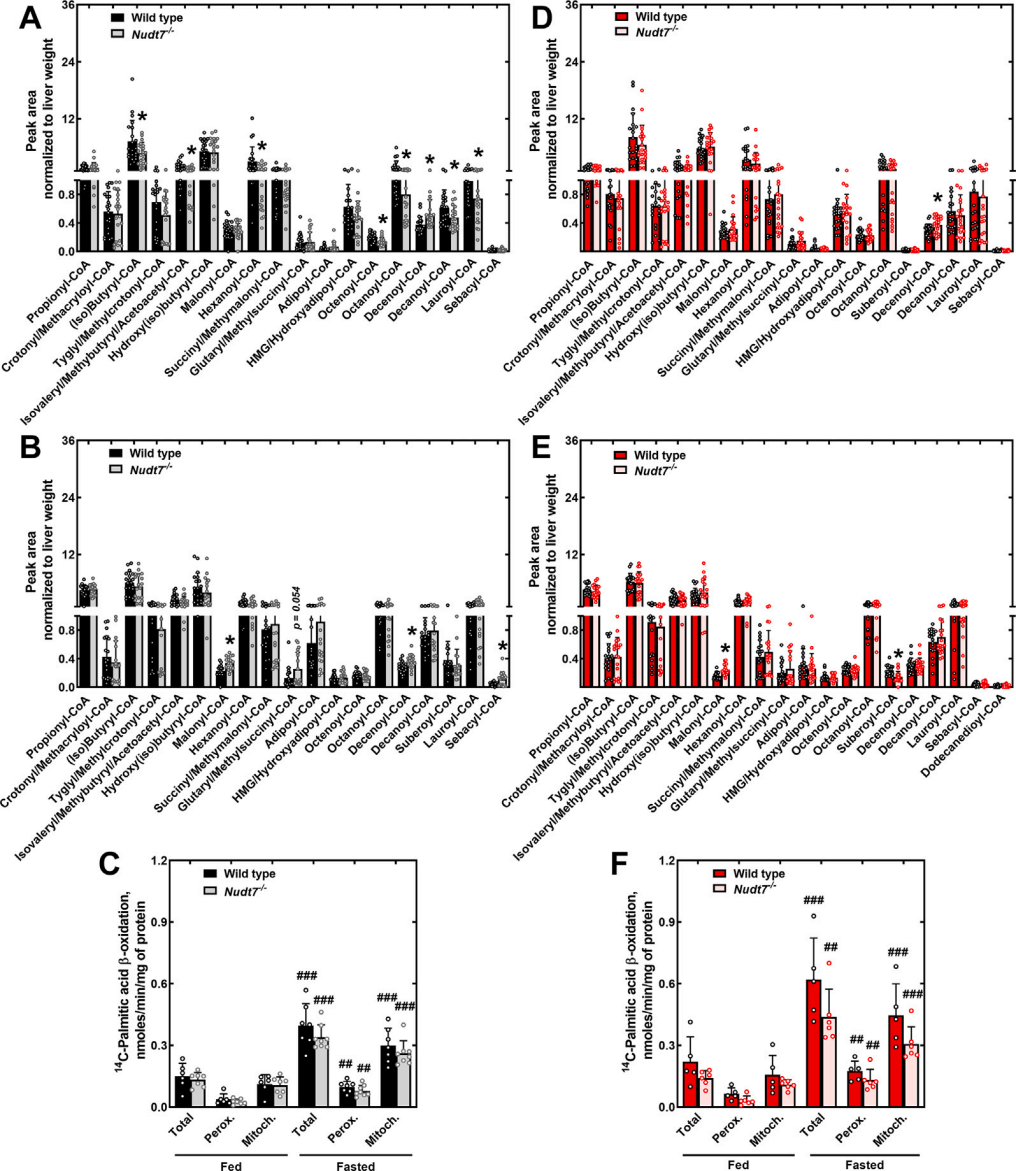
**Deletion of *Nudt7* alters the composition of the acyl-CoA pool in the liver but not the rate of fatty acid oxidation**. *A*, *B*, *D*, and *E*, liver short- and medium-chain acyl-CoA composition in male (*A* and *B*) and female (*D* and *E*) mice in the fed (*A* and *D*) or 24 h–fasted (*B* and *E*) states. Data are shown as the mean (*bar*) of measurements conducted on individual mice (*circles*) ± SD. Student’s *t* test, ∗*p* < 0.05. *C* and *F*, rate of fatty acid β-oxidation in primary hepatocytes isolated from fed or 24 h–fasted male (*C*) and female (*F*) mice in the presence or absence of etomoxir. Data are shown as the mean (*bar*) of measurements conducted on individual mice (*circles*) ± SD. Two-way ANOVA, ## *p* < 0.01, ### *p* < 0.001 between mice of the same genotype in the fed *versus* fasted state. Mitoch., mitochondrial (total – peroxisomal fatty acid oxidation); Perox., peroxisomal (+etomoxir).

Recombinant NUDT7 exhibits a broad substrate specificity, readily hydrolyzing a variety of CoA species, including short- and medium-chain acyl-CoAs (23, 24). We next determined whether deletion of *Nudt7* may have altered the composition of the hepatic acyl-CoA pool and, in particular, led to the accumulation of potential *in vivo* substrates of this enzyme (Figs. 2 and S1). In the fed state, both male and female *Nudt7*^*-/-*^ mice showed higher levels of hepatic decenoyl-CoA than control mice (Fig. 2, *A* and *D*). The liver of fed *Nudt7*^*-/-*^ males also exhibited a decrease in several short- and medium-chain acyl-CoAs. Following a 24 h fast, hepatic decenoyl-CoA remained significantly elevated in the liver of the *Nudt7*^*-/-*^ males, and sebacyl-CoA, a 10 carbon-long (C10) intermediate in the oxidation of dicarboxylic fatty acids, was also found to accumulate (Fig. 2*B*). Malonyl-CoA, whose concentration was significantly decreased in the liver of fasted mice overexpressing NUDT7 (49), was significantly elevated in the liver of both male and female *Nudt7*^*-/-*^ mice (Fig. 2, *B* and *E*), suggesting that NUDT7 activity contributes to the regulation of hepatic malonyl-CoA levels in the fasted state. Interestingly, peroxisomes are recognized sources of cytosolic malonyl-CoA (52, 53, 54), an endogenous inhibitor of carnitine palmitoyltransferase 1 (CPT1) and thus, mitochondrial fatty acid β-oxidation. Measurement of fatty acid oxidation in primary mouse hepatocytes isolated from *Nudt7*^*-/-*^ and control mice showed the expected increase in the rate of ^14^C-palmitate oxidation in hepatocytes isolated from fasted mice, compared to fed mice (55, 56) (Fig. 2, *C* and *F*). *Nudt7*-deficient hepatocytes isolated from fasted mice also tended to exhibit lower rates of mitochondrial fatty acid oxidation than WT hepatocytes, but the difference did not reach statistical significance. These results suggest that the increase in the concentration of malonyl-CoA in the liver of *Nudt7*^*-/-*^ mice was not sufficient to appreciably inhibit CPT1. Alternatively, the buildup of malonyl-CoA, potentially formed from the oxidation of odd-chain dicarboxylic fatty acids, may have been contained within the peroxisomes and thus, unable to affect the enzyme. Peroxisomal oxidation of ^14^C-palmitate, measured by inhibiting the mitochondrial β-oxidation pathway with etomoxir, was also unaffected by *Nudt7* deletion, indicating that any accumulation of acyl-CoAs would more likely occur by decreased degradation, as opposed to increased production.

### NUDT7 regulates total hepatic CoA levels in male mice fed a Western diet

**Figure 3:**
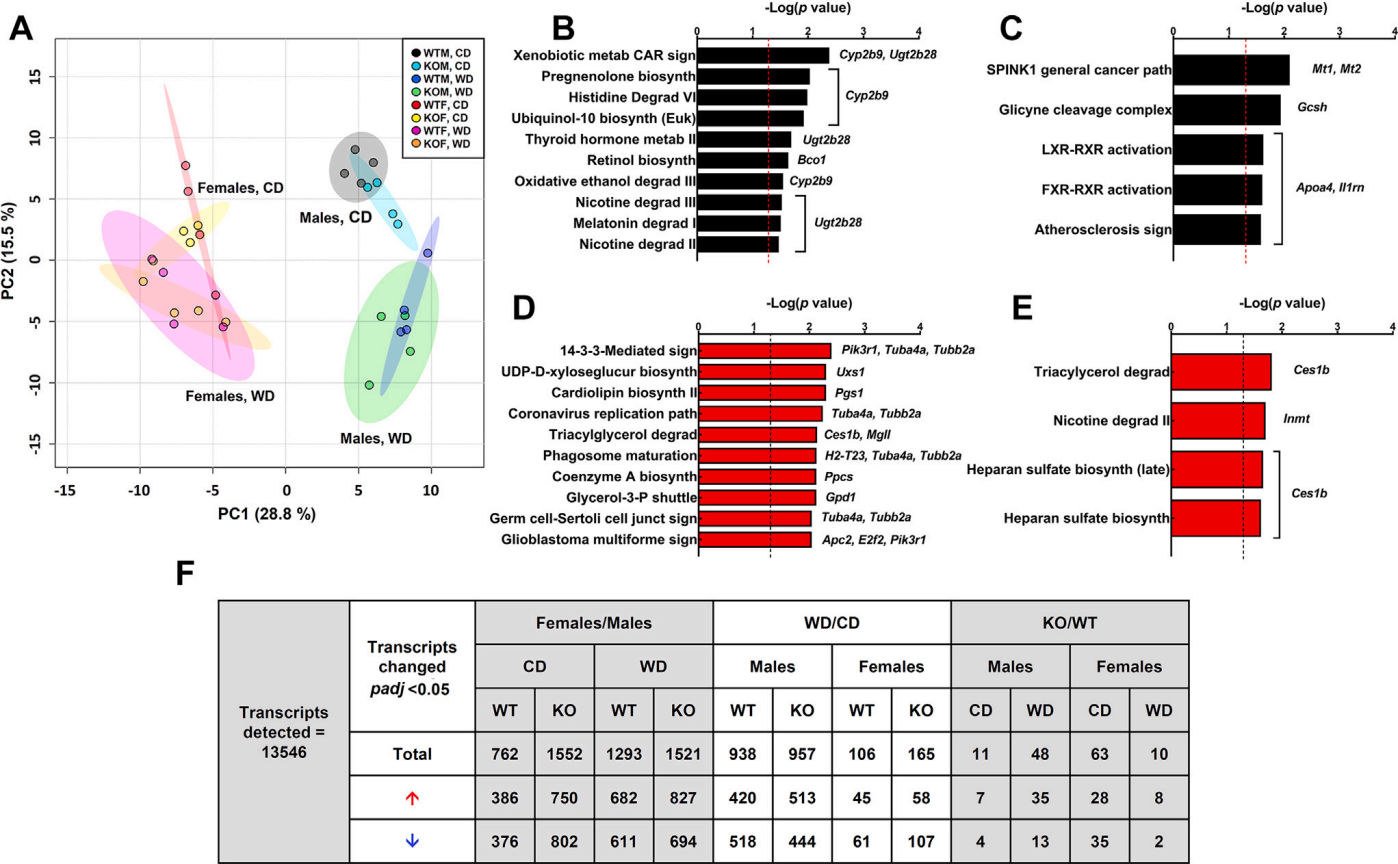
**Liver RNA-seq analysis**. *A*, principal component analysis of the RNA-seq data, with individual liver samples shown as *circles*. *B*–*E*, Ingenuity pathway analysis of the RNA-seq data showing the most significant canonical pathways affected in male (*B*) and female (*D*) mice fed the CD, and in male (*C*) and female (*E*) mice fed the WD. The gene(s) driving the difference in each pathway are listed to the *right*. Right-tailed Fisher’s Exact Test. The *dashed line* represents the threshold *p* value  of  0.05. *F*, number of transcripts differentially expressed for each indicated pairwise comparison. A false discovery rate with an adjusted *p* value (*padj*) < 0.05 is considered significant. CD, control diet; WD, Western diet.

As both peroxisomal fatty acid β-oxidation and bile acid metabolism were altered in mice with hepatic overexpression of NUDT7 (49), we fed the *Nudt7*^*-/-*^ and control mice a Western diet (WD) to challenge these pathways. Compared to mice fed the CD, mice fed the WD exhibited a higher concentration of total cholesterol in the serum and elevated triacylglycerol and total cholesterol in the liver (Table 1). RNA-seq analysis showed that, regardless of the genotype, feeding the WD changed the expression of ∼900 genes in the liver of male mice and fewer than 200 genes in the liver of female mice (Fig. 3*F*). Compared to males fed the CD, males fed the WD exhibited a global downregulation of hepatic genes involved in the uptake (*Ldlr*) and *de novo* synthesis of cholesterol, together with a robust upregulation of several genes involved in both mitochondrial and peroxisomal fatty acid oxidation (Fig. S2, *G* and *F*). These diet-induced changes in gene expression showed a similar trend in the female livers but did not reach statistical significance (Fig. S2, *J* and *I*). Only *Abcg8* and/or *Abcg5*, genes involved in cholesterol efflux, were found to be consistently upregulated in all mice fed the WD.

**Figure 4:**
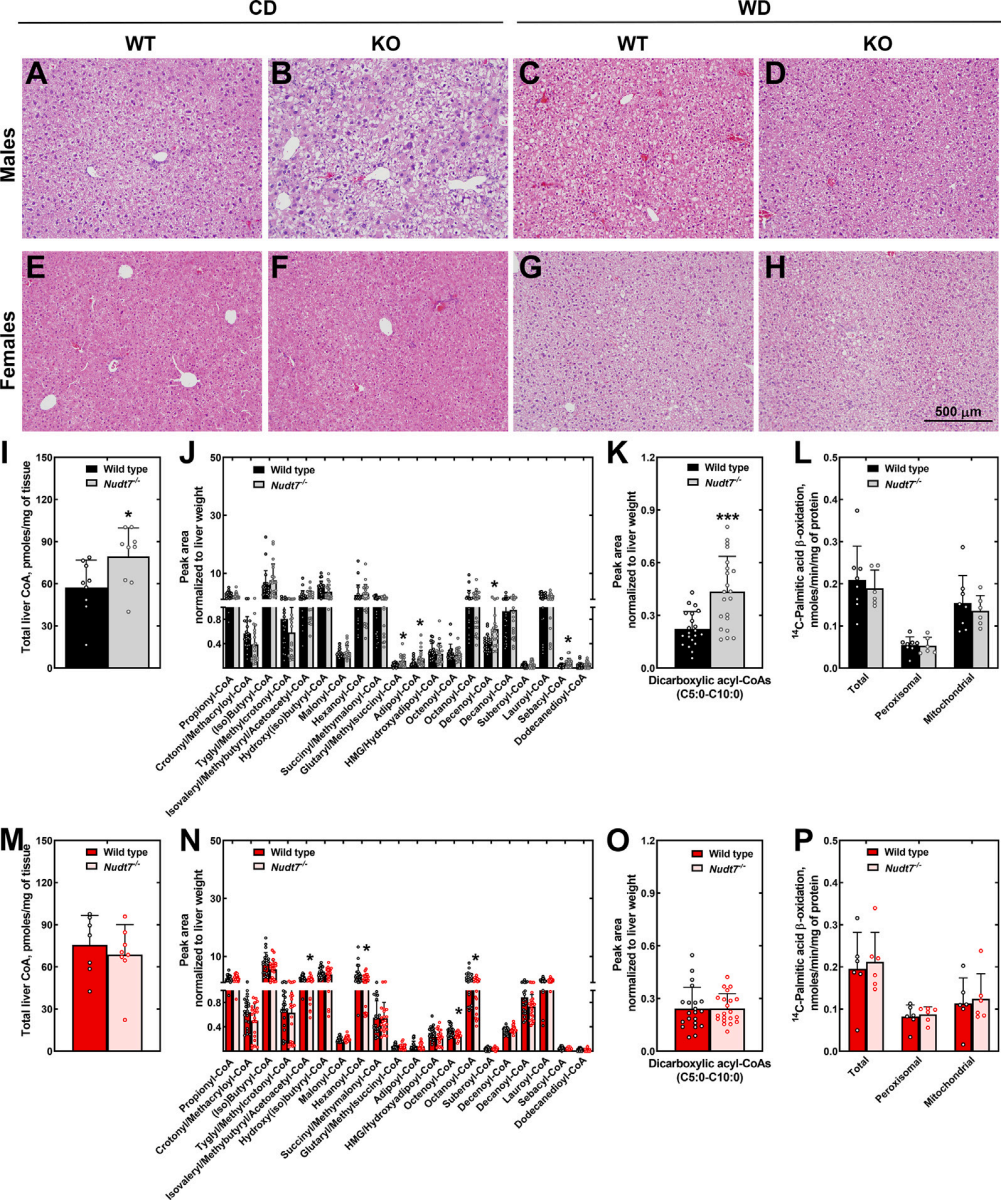
**Histological analysis and effect of WD on the liver acyl-CoA composition**. *A*–*H*, representative bright field images of H&E-stained liver sections from male (*A*–*D*) and female (*E*–*H*), WT and *Nudt7*^*-/-*^ (KO) mice fed the CD and WD. *I* and *M*, total liver CoA levels in (*I*) male and (*M*) female mice fed the WD. *J* and *N*, short- and medium-chain acyl-CoA composition of livers from (*J*) male and (*N*) female mice fed the WD. *K* and *O*, combined peak areas of C5:0-C10:0 dicarboxylic acyl-CoAs in males (*K*) and females (*O*). *L* and *P*, rate of fatty acid β-oxidation in primary hepatocytes isolated from WD-fed male (*L*) and female (*P*) mice in the presence or absence of etomoxir. Data are shown as the mean (*bar*) of measurements conducted on individual mice (*circles*) ± SD. Student’s *t* test, ∗*p* < 0.05, ∗∗∗*p* < 0.001. CD, control diet; Perox., peroxisomal (+etomoxir), Mitoch., mitochondrial (total – peroxisomal fatty acid oxidation); WD, Western diet.

Deletion of *Nudt7* altered multiple hepatic processes, as revealed by Ingenuity pathway analysis. However, these changes were driven by a very small number of differentially expressed genes (Fig. 3, *B*–*E*). In mice fed the WD, the expression of *Nudt8*, *Nudt19*, and *Fit2* was similar between genotypes (Fig. S2, *E* and *H*). Furthermore, WT mice fed the WD exhibited comparable hepatic levels of *Nudt7* transcript and protein compared to gender-matched mice fed the CD (Fig. S2, *A*–*C*, *E*, and *H*). No inflammation was observed in the liver of mice fed the WD; however, macrovesicular steatosis was consistently present, with no noticeable differences between genotypes (Fig. 4, *A*–*H*).

We proceeded with the measurement of total CoA levels in the liver of mice fed the WD. In males, deletion of *Nudt7* led to a significant increase in total hepatic CoA levels, a phenotype that was not observed in *Nudt7*^*-/-*^ females (Fig. 4, *I* and *M*). The higher concentration of total CoA in the livers of the *Nudt7*^*-/-*^ males correlated with a robust increase in glutaryl-, adipoyl- and sebacyl-CoA, MCD acyl-CoAs with 5, 6, and 10 carbons, respectively (Fig. 4, *J* and *K*). Decenoyl-CoA was also significantly elevated, similar to what was observed in *Nudt7*^*-/-*^ males fed the CD. In female mice, *Nudt7* deletion led to a decrease in multiple C4-C8 acyl-CoAs (Fig. 4*N*). These changes in acyl-CoA composition were not associated with alterations in the rate of total or peroxisomal ^14^C-palmitic acid oxidation, as measured in isolated hepatocytes (Fig. 4, *L* and *P*). Levels of other CoA species, including CoA, acetyl-CoA, and long-chain acyl-CoAs, were similar between genotypes (Fig. S1). Combined, these results support the conclusion that NUDT7 prevents the accumulation of MCD acyl-CoAs in the context of an increased influx of fatty acids, as more acutely observed when male mice were fed a WD. Under these dietary conditions, NUDT7 also regulates total CoA levels. Additionally, decenoyl-CoA was consistently elevated in male livers, regardless of the diet.

### Effect of *Nudt7* deletion on hepatic metabolism

**Figure 5:**
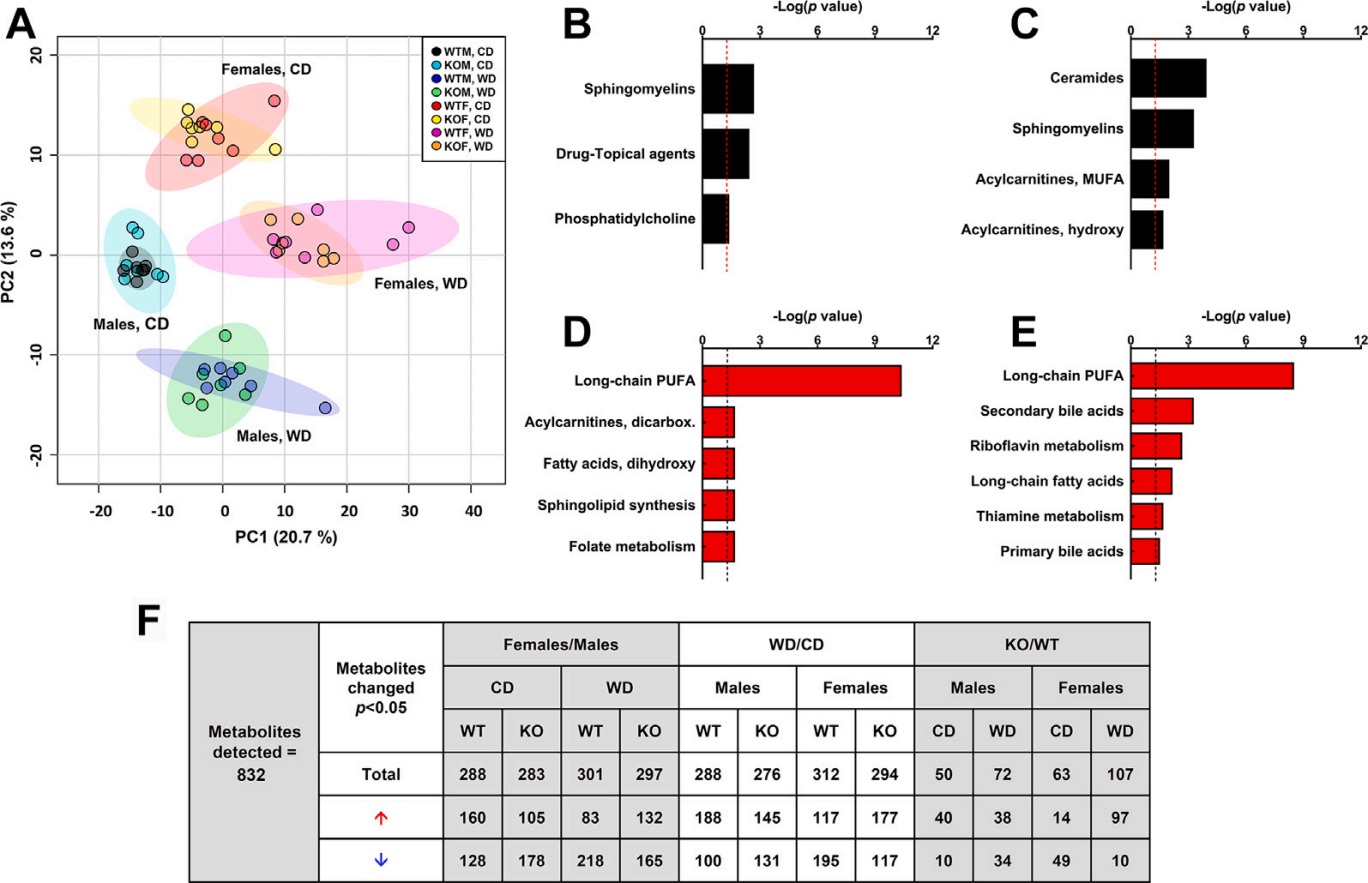
**Global changes in liver lipid metabolism**. *A*, principal component analysis of the untargeted metabolomics data, with individual liver samples shown as *circles*. *B*–*E*, pathways enriched in male (*B*) and female (*D*) mice fed the CD or in male (*C*) and female (*E*) mice fed the WD. Hypergeometric distribution test, with the *dashed line* representing the threshold *p* value  of  0.05. *F*, number of significantly changed metabolites. Three-way ANOVA with *p* value < 0.05 is considered significant. CD, control diet; WD, Western diet.

To determine whether deletion of *Nudt7* had a significant impact on liver metabolism, we conducted global metabolic profiling of WT and *Nudt7*^*-/-*^ livers isolated from mice fed the CD and the WD (Fig. 5). A total of 832 metabolites were detected in this analysis, with a larger number of metabolites being altered by gender or diet than genotype (Fig. 5*F*). In mice fed the CD, *Nudt7* deletion significantly altered the concentration of 50 metabolites in males and 63 metabolites in females. These numbers increased to 72 and 107 metabolites, respectively, in mice fed the WD. Regardless of the diet, deletion of *Nudt7* predominantly affected lipid metabolism in both males and females (Fig. 5, *B*–*E*).

In the liver of *Nudt7*^*-/-*^ males fed the CD, a number of sphingomyelin, phosphatidylcholine, and lysophospholipid species were significantly increased, compared to control males (Table S2). This trend was inverted in *Nudt7*^*-/-*^ males fed the WD, as their livers contained lower levels of several sphingomyelins while accumulating multiple ceramides. Consistent with the increase in glutaryl-CoA, the livers of *Nudt7*^*-/-*^ males fed the WD showed a 60% increase in the concentration of glutarylcarnitine, together with other short-chain and MCD acylcarnitines, including succinoylcarnitine, methylsuccinoylcarnitine, and pimeloyl/3-methyladipoylcarnitine. Conversely, the levels of several long-chain acylcarnitines were significantly lower in the *Nudt7*^*-/-*^ livers, and this correlated with a trend toward a decreased concentration of free carnitine.

The livers of *Nudt7*^*-/-*^ females showed highly significant changes in long-chain polyunsaturated fatty acids on both diets (Fig. 5, *D* and *E*). In *Nudt7*-deficient livers isolated from females fed the CD, ω-3 and ω-6 fatty acids were decreased by about 50%, including the essential fatty acids linoleate and linolenate (Table S2). The trend toward a decrease extended to endogenously synthesized fatty acids; however, with the exception of oleate, the differences between *Nudt7*^*-/-*^ and WT livers did not reach statistical significance. In WT females, the WD led to a selective and pronounced 50 to 90% decrease in the concentration of several ω-6 fatty acids, compared to the CD. This WD-induced decrease was not systematically observed in the livers of the *Nudt7*^*-/-*^ females, leading to an overall elevation in the levels of polyunsaturated fatty acids and several saturated and monounsaturated long-chain fatty acids, compared to control females (Table S2). Interestingly, Ingenuity pathway analysis of the RNA-seq data identified triacylglycerol degradation as a process altered by *Nudt7* deletion in female mice fed either diet (Fig. 3, *D* and *E*). Indeed, *Ces1b*, a member of the promiscuous *CES1* carboxylesterase family (57, 58), was consistently upregulated ∼7-fold in the liver of *Nudt7*^*-/-*^ females, regardless of the diet. The liver of *Nudt7*^*-/-*^ females fed the CD also exhibited a significant downregulation in the gene encoding monoglyceride lipase, *Mgll*, and a strong trend toward increased triacylglycerol excretion in the feces (Fig. 7*G*), which could contribute to the diminished concentration of nonesterified fatty acids in the liver of these mice. The untargeted metabolomics analysis also revealed that the *Nudt7*^*-/-*^ females fed the WD contained altered levels of both primary and secondary bile acids (Table S2). As overexpression of NUDT7 had the opposite effect in the liver of fasted mice (49), these results strengthened the connection between NUDT7 activity and bile acid metabolism. Altogether, the global metabolic profiling data revealed that *Nudt7* deletion affected hepatic lipid metabolism in both male and female mice, with specific pathways differing between genders.

**Figure 6:**
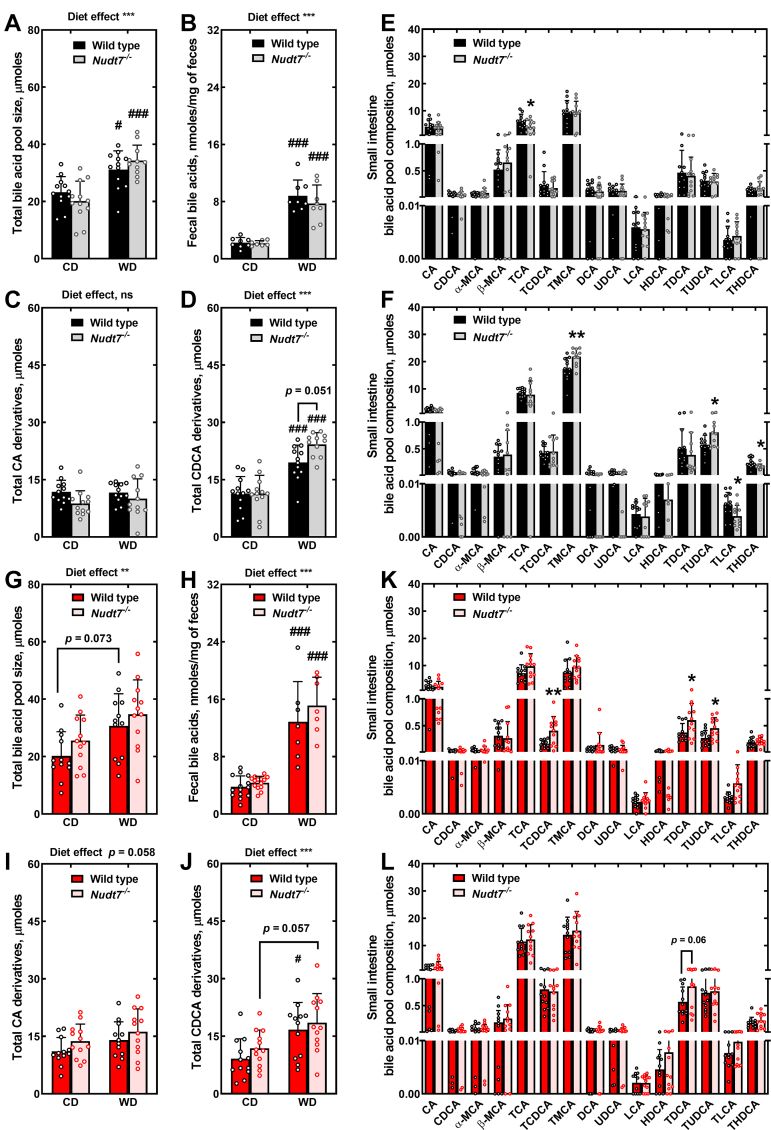
**Deletion of *Nudt7* alters the composition of the intestinal bile acid pool**. *A* and *G*, total bile acid pool sizes in male (*A*) and female (*G*) mice. *B* and *H*, concentration of fecal bile acids in male (*B*) and female (*H*) mice. *C* and *I*, total CA derivatives combined across the liver, gallbladder, and small intestine of (*C*) male and (*I*) female mice. *D* and *J*, total CDCA derivatives combined across the liver, gallbladder, and small intestine of (*D*) male and (*J*) female mice. Data are shown as the mean (*bar*) of measurements conducted on individual mice (*circles*) ± SD. Two-way ANOVA, # *p* < 0.05, ### *p* < 0.001 between mice of the same genotype–fed different diets, ∗∗*p* < 0.01, ∗∗∗*p*< 0.001 for diet effect, ns, not significant. *E* and *K*, bile acid composition of the small intestine of males (*E*) and females (*K*) fed the CD. *F* and *L*, bile acid composition of the small intestine of males (*F*) and females (*L*) fed the WD. Data are shown as the mean (*bar*) of measurements conducted on individual mice (*circles*) ± SD. Student’s *t* test, ∗*p* < 0.05, ∗∗*p* < 0.01. CA, cholic acid; CDCA, chenodeoxycholic acid; CD, control diet; WD, Western diet.

**Figure 7:**
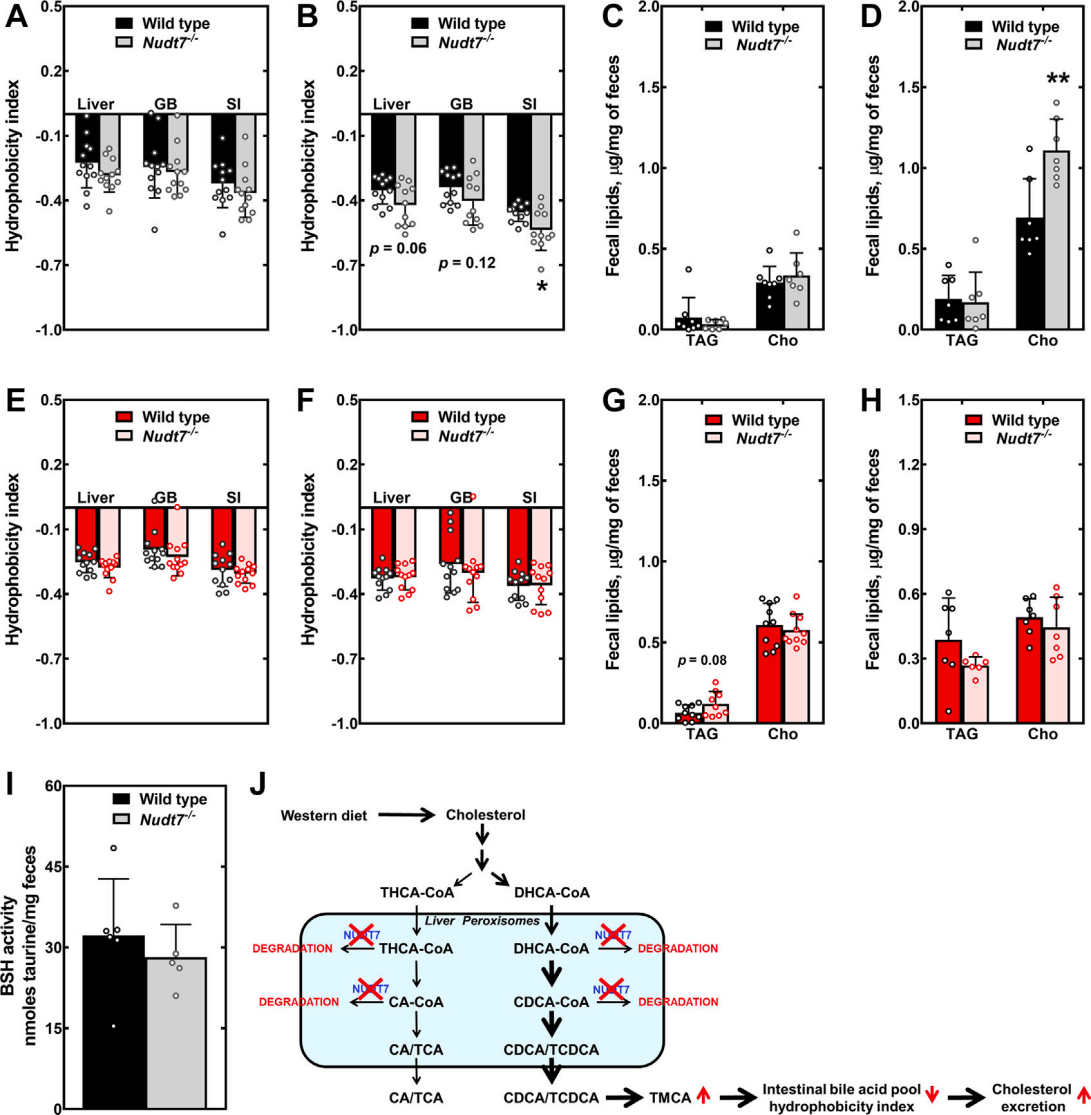
**Deletion of *Nudt7* decreased the hydrophobicity index of the intestinal bile acid pool and increases fecal cholesterol excretion in males fed the WD**. *A* and *E*, hydrophobicity index of the bile acid pools in males (*A*) and females (*E*) fed the CD. *B* and *F*, hydrophobicity index of the bile acid pools in males (*B*) and females (*F*) fed the WD. *C* and *G*, triacylglycerol (TAG) and cholesterol (Cho) levels in the feces of males (*C*) and females (*G*) fed the CD. *D* and *H*, triacylglycerol (TAG) and cholesterol (Cho) levels in the feces of males (*D*) and females (*H*) fed the WD. *I*, fecal BSH activity measured in males fed the WD. Data are shown as the mean (*bar*) of measurements conducted on individual mice (*circles*) ± SD. Student’s *t* test, ∗*p* < 0.05, ∗∗*p* < 0.01. *J*, effect of *Nudt7* deletion on the synthesis of TMCA and fecal cholesterol excretion in males fed the WD. BSH, bile salt hydrolase; CD, control diet; GB, gallbladder; SI, small intestine; TMCA, tauromuricholic acid; WD, Western diet.

### NUDT7 regulates the composition of the intestinal bile acid pool and the fecal excretion of cholesterol in male mice fed the WD

To gain further insight into the role that NUDT7 plays in the regulation of bile acid metabolism, we used a targeted analysis to quantify multiple primary and secondary bile acids in the liver, gallbladder, and small intestine of mice fed the CD and the WD (Figs. 6 and S3). Combining the concentration of all detected bile acids across these organs provided an estimate of the total bile acid pool size in each mouse. As expected, the relatively high cholesterol content of the WD diet increased the size of the total bile acid pool in both males and females (59, 60), with no differences between genotypes (Fig. 6, *A* and *G*). This was associated with a robust accumulation of the bile acid precursor 7α-hydroxy-4-cholesten-3-one (7α-hydroxycholestenone or C4) in the serum and liver of mice fed the WD (Tables 1 and S2) and with a ∼ 3-fold increase in the fecal excretion of bile acids (Fig. 6, *B* and *H*), regardless of gender and genotype. These results supported the conclusion that the WD-induced expansion of the total bile acid pool was due to increased *de novo* synthesis of these compounds in the liver, as opposed to decreased excretion. As serum C4 levels are considered a marker for the activity of CYP7A1 (61), the rate-limiting enzyme in the classic bile acid biosynthetic pathway, these results also indicated that feeding the WD increased the activity of this enzyme, independent of changes in transcript levels (Fig. S4, *C* and *F*).

The two direct products of the bile acid biosynthetic pathways in mice are CA, CDCA, and their taurinated derivatives, which are then converted to other primary and secondary species in the liver and intestine, respectively. By separately plotting the total amount of CA and CDCA derivatives, we found that the diet had a significant effect on the concentration of CDCA-derived bile acids, leading to a ∼2-fold increase in mice fed the WD (Fig. 6, *C*, *D*, *I*, and *J*). In WT males, feeding the WD was also associated with a significant downregulation of hepatic *Cyp8b1*, which could account for the diet-induced change in bile acid pool composition (Fig. S4*C*). In the *Nudt7*^*-/-*^ males fed the WD, the accumulation of CDCA derivatives was even higher than in WD-fed control males, narrowly missing statistical significance, and independent of differences in the expression of genes involved in bile acid synthesis (Figs. 6*D* and S4*C*). The WD-induced changes in the concentration of CDCA-derived bile acids in female mice were overall more modest than those observed in males, with no differences between genotypes (Fig. 6*J*). Combined, these results indicated that *Nudt7* deletion did not affect the size of the total bile acid pool. However, under condition of increased bile acid synthesis, lack of NUDT7 activity exacerbated the accumulation of CDCA derivatives in male mice.

We next examined the composition of the individual bile acid pools. In mice fed the CD, deletion of *Nudt7* had no significant effect on the concentration of the bile acids detected in the liver and gall gladder, regardless of the gender (Fig. S3, *A*, *D*, *G*, and *J*). However, deletion of *Nudt7* was associated with a significant ∼25% drop in the concentration of intestinal taurocholic acid (TCA) in males and with the accumulation of taurochenodeoxyholic acid (TCDCA), taurodeoxycholic acid (TDCA), and tauroursodeoxycholic acid (TUDCA) acid in the intestine of female mice (Fig. 6, *E* and *K*). When fed the WD, both the liver and the small intestine of the *Nudt7*^*-/-*^ males exhibited a significant ∼25% increase in tauromuricholic acid (TMCA), one of the major primary bile acids in mice that derives from CDCA (Figs. 6*F* and S3*B*). In mice, TUDCA is considered to be a CDCA-derived primary bile acid (62), and the concentration of this compound was also significantly elevated in the small intestine of the WD-fed *Nudt7*^*-/-*^ males (Fig. 6*F*). Consistent with the net increase in the concentration of CDCA derivatives observed in these mice, the accumulation of TMCA and TUDCA was not compensated for by the ∼30% decrease in the concentration of the secondary bile acids taurolithocholic acid (TLCA) and taurohyodeoxycholic acid (THDCA), which are minor components of the hepatic and intestinal bile acid pools. As the bile acids move through the intestine, a portion of these compounds is deconjugated by the microbial enzyme bile salt hydrolase (BSH), a step that is required for their further metabolism by the gut microbiome and their conversion to secondary bile acids. To determine whether accumulation of TMCA and TUDCA in the *Nudt7*^*-/-*^ mice could be due to a decrease in deconjugation activity, we measured BSH activity in fecal extracts and found no differences between genotypes (Fig. 7*I*). Combined, these results suggested that *Nudt7* deletion further enhanced the synthesis of CDCA-derived primary bile acids already stimulated by WD feeding in male mice.

*Nudt7*^*-/-*^ females fed the WD did not show, by our targeted analysis, the extensive changes in hepatic bile acid levels detected by the untargeted metabolomics analysis. An exception was the robust 3-fold increase in the concentration of hepatic deoxycholic acid (DCA) compared to control females, which was detected by both analyses (Table S2 and Fig. S3*H*). Our targeted bile acid analysis was conducted on a larger number of mice and optimized for the extraction, separation, and quantitation of these metabolites, factors that likely account for the discrepancy between methodologies. No significant changes were found in the composition of the intestinal bile acid pool between genotypes, although TDCA tended to be higher in WD-fed *Nudt7*^*-/-*^ females than WT females (Fig. 6*L*). In both males and females fed the WD, the composition of the gallbladder bile acid pool was not significantly different between genotypes (Fig. S3, *D*, and *J*). These results indicated that deletion of *Nudt7* led to gender-specific perturbations in the composition of the hepatic and intestinal bile acid pools in mice fed a WD. In the small intestine, differences in bile acid pool composition were also observed in *Nudt7*^*-/-*^ mice fed the CD.

Bile acids can feed-back inhibit their own synthesis by binding to the nuclear receptor FXR in the liver and intestine with varying affinities (62, 63, 64, 65). To determine whether the observed alterations in intestinal bile acid composition affected FXR activity, we measured the transcript levels of two known FXR target genes: *Fgf15* and *Shp*. TCDCA and TDCA are potent FXR agonists (63, 64, 65). Consistent with the accumulation of these bile acids, we found the *Fgf15* transcript levels in the intestine of *Nudt7*^*-/-*^ females fed the CD to be significantly higher than WT females (Fig. S4*D*). Similarly, the decrease in the concentration of TCA, another FXR agonist, was mirrored by the downregulation of *Fgf15* in the intestine of *Nudt7*^*-/-*^ males fed the CD (Fig. S4*A*). In contrast, the increase in the concentration of TMCA, an FXR antagonist, was not sufficient to elicit changes in *Fgf15* mRNA levels in the intestine of *Nudt7*^*-/-*^ males fed the WD (Fig. S4*B*). Intestinal *Shp* expression was similar between genotypes, regardless of diet. In the liver, activation of FXR by bile acids cooperates with the action of intestine-derived FGF15/19 to blunt bile acid synthesis through the downregulation of *Cyp7a1* and *Cyp8b1* (66, 67, 68). However, neither of these two genes was differentially expressed between genotypes, regardless of the diet (Fig. S4, *C* and *F*), indicating that while the changes in bile acid composition in the *Nudt7*^*-/-*^ mice had some effect on intestinal FXR activity in mice fed the CD, they had no overt effect on the expression of genes involved in the synthesis of bile acids in the liver.

The composition of the bile acid pool can affect intestinal lipid absorption (45, 69, 70). To determine whether the changes in bile acid composition observed in the *Nudt7*^*-/-*^ mice altered the hydrophilic-hydrophobic balance of the bile acid pools, we calculated their hydrophobicity index (Fig. 7, *A*, *B*, *E*, and *F*). We found that the hydrophobicity index of the intestinal bile acid pool was significantly decreased in the *Nudt7*^*-/-*^ males fed the WD (Fig. 7*B*). In these mice, the hydrophobicity index of the liver bile acid pool also tended to be lower than that of control mice, without reaching statistical significance. In the liver and intestine, this effect was mainly driven by the higher molar fraction of TMCA, a very hydrophilic bile acid. The lower hydrophobicity index of the intestinal bile acid pool correlated with a robust increase in the concentration of cholesterol excreted in the feces of the *Nudt7*^*-/-*^ mice fed the WD, with no difference in the levels of triacylglycerols (Fig. 7*D*). The hydrophobicity index of the liver, gallbladder, and small intestine bile acid pools remained comparable between genotypes in female mice, regardless of the diet (Fig. 7, *E* and *F*). Consistent with this finding, the amount of cholesterol excreted in the feces was similar between WT and *Nudt7*^*-/-*^ females (Fig. 7, *G* and *H*). Taken together, these results support the conclusion that, in the context of increased cholesterol intake and bile acid synthesis, the (acyl-)CoA-degrading activity of NUDT7 contributes to the regulation of intestinal cholesterol absorption by modulating the composition of the bile acid pool in male mice (Fig. 7*J*).

## Discussion

While four enzymes are now known to hydrolyze CoA species at the phosphodiester bond in different organs and subcellular organelles, the physiological functions and metabolic processes regulated by these enzymes are still poorly characterized (21, 22, 25, 26). Having previously shown that liver-specific overexpression of NUDT7 was not sufficient to prevent the increase in total hepatic CoA levels that occurs in fasted mice (49), a primary goal of this study was to determine whether the lack of this peroxisomal enzyme could regulate total CoA levels under conditions, such as the fed state, when hepatic NUDT7 protein levels are at their highest (18, 24). We found that deletion of *Nudt7* in mice fed the CD decreased the total CoA-degrading activity detected in liver homogenates by 80%; however, this dramatic drop in activity was not associated with differences in total hepatic CoA levels between genotypes, regardless of the nutritional state. Combined with the overexpression study (49), these findings clearly show that the activity of NUDT7 does not contribute to the changes in total hepatic CoA levels that occur between the fed and fasted states (16, 17, 18). These results also support the conclusion that, in spite of the abundance of NUDT7 in the liver, the hydrolytic activity of this enzyme may be limited by the substrate availability inside the peroxisomes, which are estimated to contain only a small percentage of the total cellular CoA (11, 12). It remains to be determined which of the other CoA-degrading enzymes, alone or in combination, is responsible for lowering total hepatic CoA levels during the transition from the fasted state to the fed state. As NUDT19 levels are hardly detectable in mouse liver under these conditions (24), NUDT8 and FIT2 are the most likely candidates (25, 26). We also cannot rule out the existence of additional, currently unknown enzymes with the ability to break down CoA species.

Unlike what we observed in mice fed the CD, *Nudt7* deletion led to a robust increase in total hepatic CoA levels in male mice fed the WD, an effect that was primarily driven by the gender-specific accumulation of MCD acyl-CoAs (Fig. 4, *I*–*K*). These metabolites can be generated by the shortening of long-chain (≥C12) dicarboxylic fatty acids, a process that preferentially occurs in the peroxisomes by the action of the same set of enzymes that oxidize palmitic acid (33, 34, 71, 72, 73, 74). Long-chain dicarboxylic fatty acids are formed from the correspondent monocarboxylic fatty acids through oxidation of the terminal methyl group. This process, called ω-oxidation, is initiated by microsomal enzymes that belong to the CYP4A family (75, 76, 77) and is induced under conditions that increase the fatty acid supply to the liver (30, 31, 32, 78). Compared to mice fed the CD, feeding the WD led to a significant, male-specific upregulation of *Cyp4a* genes involved in ω-oxidation (32, 79) in the liver of both WT and *Nudt7*^*-/-*^ mice (Fig. S2*F*). The most dramatic effect was observed in the *Nudt7*^*-/-*^ males, which showed a 12-fold and 15-fold upregulation of *Cyp4a10* and *Cyp4a14*, respectively (Fig. S2*F* and Table S3). NUDT7 transcript and protein levels were also significantly more abundant in the liver of male mice than female mice (Figs. 1, *D*, *E* and S2, *A*–*C*), consistent with previous studies investigating gender-specific hepatic gene expression (80, 81) and with the higher total CoA-degrading activity in liver homogenates obtained from male mice (Fig. 1*C*). The higher concentration of NUDT7, combined with the upregulation of the peroxisomal fatty acid oxidation pathway and the males-specific upregulation of *Cyp4a* genes, could explain the accumulation of MCD acyl-CoAs in the liver of *Nudt7*^*-/-*^ male, but not female, mice fed the WD. Under these conditions, the increase in the concentration of MCD acyl-CoAs occurred without affecting the rate of their production through fatty acid β-oxidation, strongly suggesting that these metabolites are NUDT7 substrates *in vivo*. Interestingly, enzymes that could compete for the same substrates within the peroxisomes, such as carnitine octanoyltransferase, CROT, and acyl-CoA thioesterases ACOT4, and ACOT8 (29, 82), were not able to prevent the accumulation of MCD acyl-CoAs in the liver of the *Nudt7*^*-/-*^ mice, supporting the conclusion that degradation by NUDT7 is an important step in the downstream metabolism of these products.

In addition to MCD acyl-CoAs, decenoyl-CoA and malonyl-CoA were also found to accumulate in the liver of both male and female *Nudt7*^*-/-*^ mice under different conditions (Fig. 2, *A*, *B*, *D*, and *E*). Decenoyl-CoA can be formed in the peroxisomes as an intermediate product of the fatty acid β-oxidation pathway (73). Interestingly, degradation of decenoyl-CoA by NUDT7 would halt the oxidation of a fatty acid at the 10 carbon length, potentially contributing to the known acyl-chain shortening, as opposed to full oxidation, that occurs in these organelles. The buildup of malonyl-CoA in the liver of both male and female *Nudt7*^*-/-*^ mice was detectable only in the fasted state. Depending on the mode and site of its production under these conditions, malonyl-CoA may or may not be a direct substrate for NUDT7 *in vivo*. Indeed, malonyl-CoA could be produced by the complete oxidation of odd-chain dicarboxylic fatty acids within the peroxisomes or by the re-activation of peroxisome-derived acetate to acetyl-CoA in the cytosol, followed by carboxylation. Based on the fact that the accumulation of malonyl-CoA did not significantly affect the rate of mitochondrial fatty acid oxidation in primary hepatocytes (Fig. 2, *C* and *F*) and that the activity of acetyl-CoA carboxylase is expected to be low in the fasted state (83), we currently deem more likely that malonyl-CoA accumulated within the peroxisomes due to the lack of NUDT7-mediated *in situ* degradation. Obtaining direct proof would require the measurement of the acyl-CoA pool composition in both cytosol- and peroxisome-enriched subcellular fractions. Major technical improvements have now made this type of analysis possible in cytosolic-, mitochondrial-, and nuclear-enriched fractions (14, 84, 85, 86); however, the isolation of intact peroxisomes that are uncontaminated by other organelles remains a challenge (25, 87). Accumulation of malonyl-CoA in immature murine articular chondrocytes isolated from an independently derived *Nudt7*^*-/-*^ mouse model further support a role for NUDT7 activity in the regulation of this metabolite (50).

The second major goal of this study was to gain insight into the role that NUDT7 plays in the regulation of liver metabolism. Consistent with the key role that peroxisomes play in lipid metabolism, untargeted metabolomics revealed that *Nudt7* deletion affected the concentration of fatty acids, acylcarnitines, bile acids, sphingolipids, and ceramides (Fig. 5, *B*–*E*). We then focused our targeted analysis on peroxisomal fatty acid β-oxidation and bile acid metabolism. Both these processes were blunted in the liver of mice overexpressing NUDT7 (49) and could have been linked to the changes in fatty acids, acyl-carnitines, and bile acids observed in the livers of the *Nudt7*^*-/-*^ mice. In mice with liver-specific overexpression of NUDT7, the decrease in the rate of peroxisomal fatty acid oxidation was associated with a trend toward lower levels of hepatic free CoA. This NUDT7 substrate is required for each step of fatty acid oxidation and its concentration could have been locally decreased by increased degradation in the peroxisomes. In the *Nudt7*^*-/-*^ mice, deletion of *Nudt7* did not change the rate of hepatic peroxisomal fatty acid β-oxidation, regardless of the diet and nutritional state. These results suggest that deletion of *Nudt7* had either no effect on the local concentration of CoA or that a potential increase in peroxisomal CoA was unable to accelerate reactions whose rate was set by enzyme activity. Importantly, these results indicate that the decrease in NUDT7-driven acyl-CoA degradation that occurs with fasting in WT livers does not contribute to the increased rate of fatty acid β-oxidation that occurs from the fed to the fasted state (56, 88). Interestingly, knockdown of *NUDT7* in chondrocytes decreases catalase activity and the number of peroxisomes, leading to lipid accumulation (50). We did not observe either lipid accumulation or differences in peroxisomal fatty acid oxidation in the liver of *Nudt7*^*-/-*^ mice (Table 1, Figs. 2 and 4), suggesting a potential tissue-specific function for this enzyme. While this article was under review, Song J. *et al.* reported that their independently generated, chow-fed *Nudt7*^*+/-*^ and *Nudt7*^*-/-*^ mice accumulated triacylglycerols in the liver. The *Nudt7*^*+/*-^ mice also contained a higher hepatic content of palmitate and elevated transcript levels of PPARγ target genes such as *Cd36*, *Fabp4*, and *Il6* (89). While we did not observe any of these changes, the animals we studied were significantly younger than the 12 month-old *Nudt7*^*+/-*^ and *Nudt7*^*-/-*^ mice described by Song J. *et al.* Age-dependent differences in hepatic metabolism, including accumulation of triacylglycerols, are well documented (90, 91, 92); thus, the findings by Song J. *et al.* raise the intriguing possibility that deletion of *Nudt7* may exacerbate the metabolic changes observed in aging mice.

As mentioned above, deletion of *Nudt7* had no effect on hepatic fatty acid β-oxidation; however, eliminating the activity of this enzyme changed the composition of the total bile acid pool, an effect that was more pronounced in male *Nudt7*^*-/-*^ mice fed the WD. Bile acid synthesis in the liver involves at least 17 different enzymes and multiple subcellular compartments, including the cytosol, mitochondria, ER, and peroxisomes, where the synthesis of CA, CDCA, and their taurinated derivatives is completed (40, 41). Both composition and size of the total bile acid pool are important for the regulation of glucose and lipid metabolism, and the opposite diurnal rhythm and fast/feeding regulation exhibited by CYP7A1 and CYP8B1 underscores the importance of balancing the total amount of bile acids with the appropriate ratio of hydrophilic to hydrophobic bile acids (44, 93). Indeed, bile acid composition can affect intestinal lipid absorption and signaling through FXR and G protein–coupled bile acid receptor 1 (TGR5) (94). In mice, CDCA is efficiently hydroxylated to α- and β-muricholic acid (MCA) by CYP2C70 (95, 96), and TMCAs are major, hydrophilic components of the total bile acid pool. Our data showed that the high cholesterol content of the WD resulted in the expansion of bile acid pool driven by the selective accumulation of CDCA derivatives. In males, this effect was more robust and, interestingly, correlated with 7- to 9-fold higher transcript levels of *Cyp7b1*, a key enzyme in the alternative bile acid biosynthetic pathway that yields CDCA and is known to be expressed in a gender-specific manner (Table S3) (46, 81, 97). Under these conditions, deletion of *Nudt7* exacerbated the WD-induced effect, resulting in the accumulation of TMCA in both the liver and small intestine of the *Nudt7*^*-/-*^ male mice. These results suggest that, when the flux through the bile acid biosynthetic pathway increases, NUDT7-dependent peroxisomal degradation of bile acid intermediates in the liver blunts the production of CDCA and its downstream conversion to TMCA. Thus, under these conditions, hepatic NUDT7 contributes to the regulation of the composition of the bile acid pool by modulating substrate availability for the last, peroxisomal steps in the bile acid biosynthetic pathway. Evidence supporting the conclusion that this phenotype is driven by the liver include the very high expression levels of NUDT7 in this organ (Fig. 1*B*), the selective increase in two primary bile acids TMCA and TUDCA (Fig. 6*F*), and the fact that the BSH activity was similar between genotypes (Fig. 7*I*). Modest changes in the hepatic and intestinal concentration of the secondary bile acids hyodeoxycholic acid (HDCA), THDCA, and TLCA in male mice fed the WD (Figs. 6*F* and S3*B*) also suggest that deletion of *Nudt7* might alter the 7-dehydroxylase activity of the gut microbiome, a multi-step process catalyzed by bile-acid inducible (*bai*) genes (98, 99).

Recombinant NUDT7 efficiently hydrolyzes THCA-CoA and CA-CoA (23). The effect that overexpression (49) or removal of this enzyme has on the bile acid composition of the liver strongly supports the conclusion that NUDT7 can also hydrolyze DHCA-CoA and/or CDCA-CoA. This activity, however, becomes consequential only under conditions of increased synthesis of these substrates, suggesting that, in male mice fed the CD, NUDT7 cannot compete with BAAT and ACOT8, enzymes that convert these acyl-CoAs to CDCA, CA, and their taurine conjugates (29, 100).

While TCA, a major component of the total bile acid pool, promotes cholesterol absorption, TMCA is a more hydrophilic bile acid, which can lower the capacity of the mixed micelles in the intestinal lumen to solubilize cholesterol, increasing the fecal excretion of this lipid. This has been reported, for example, in mice fed MCAs or over-producing them due to knockdown or deletion of *Cyp8b1*, which severely decrease or eliminate the production of TCA (45, 70, 101, 102). Consistent with the decrease in the hydrophobicity index of the intestinal bile acid pool, we observed increased cholesterol excretion in the *Nudt7*^*-/-*^ males fed the WD. This effect, however, was not sufficient to significantly change liver and serum total cholesterol levels (Table 1). Interestingly, as humans do not synthesize MCAs, inhibition of NUDT7 may result in higher concentrations of CDCA and conjugated derivatives, which could elicit some beneficial effects on lipid and glucose metabolism through activation of FXR and/or TGR5, following conversion of CDCA to lithocholic acid (LCA) (94, 103, 104). In female *Nudt7*^*-/-*^ mice fed the CD, the higher intestinal concentration of TCDCA was indeed associated with FXR activation and with a trend toward increased fecal excretion of triacylglycerol (Figs. 7*G* and S4*D*) (105).

The composition of the bile acid pool is also linked to the risk of developing colorectal cancer (CRC) (106, 107, 108). Thus, the ability of NUDT7 to regulate the synthesis of CDCA derivatives may also explain the recently recognized association between this enzyme and CRC. Indeed, *NUDT7* has been identified as a candidate susceptibility gene for familial CRC, with an early truncation, p.Y37X, found in two patients (109). Additionally, *Nudt7*^*-/-*^ mice treated with a combination of azoxymethane and dextran sulfate sodium develop more polyps and adenocarcinoma than WT mice (51). The changes in bile acid composition that stimulate the development of CRC have recently been linked to their effect on FXR activity (107), and deletion of *Nudt7* affected FXR activity in a diet- and gender-specific manner (Fig. S4). Thus, while our studies have identified NUDT7 as a novel player in the regulation of the composition of the bile acid pool, they also suggest that the extent to which modulating the activity of this enzyme would be beneficial will be highly context dependent.

## Experimental procedures

Reagents were purchased from the following suppliers: GAPDH and catalase antibodies from Cell Signaling Technologies; horseradish peroxidase–conjugated goat anti-rabbit secondary antibody from Thermo Fisher Scientific; bile acid standards from CDN Isotopes, Cambridge Isotope Labs, and Avanti Polar Lipids; MS-grade acetonitrile from Honeywell; TRI Reagent from Molecular Research Center, Inc.; ^14^C-palmitic acid from American Radiolabeled Chemicals, and etomoxir from Tocris Bioscience. The NUDT7 antibody was generated as described previously (18). All other chemicals were of analytical grade or better and were purchased from Sigma-Aldrich or Thermo Fisher Scientific, unless stated otherwise.

### Generation of the *Nudt7*^*-/-*^ mice and animal studies

Whole-body *Nudt7*^*-/-*^ mice were generated by CRISPR/Cas9 genome editing, which resulted in the deletion of exons 3 and 4 in the process of flanking this DNA region with LoxP sites. Single guide RNAs (5g, 5′-TCCCAACCCAACATAGTGGAAGG-3′ and 3g, 5′-TGGAATTACTCCCTAACCCCAGG-3′, protospacer adjacent motif sequences underlined) were selected and provided in a microinjection cocktail by Applied StemCell. The microinjection cocktail, which also included Cas9 mRNA and single-stranded donor oligonucleotides containing LoxP sequences and *NdeI* restriction sites, was injected into FVB mouse zygotes by the WVU Transgenic Core Facility. We obtained a single mosaic founder containing WT, floxed, and KO alleles (Fig. 1*A*). Breeding of the founder with a C57BL/6J mouse allowed us to isolate *Nudt7*^*+/-*^ mice. Amplification and sequencing of a 330 bp band obtained by multiplex PCR analysis using the Accustart II PCR Genotyping Kit (QuantaBio) and primers 5tf (5′-AGTGGCACACACTACACAAACA-3′), ko (5′-GGTGATCAAACTCAGAACCGTATGC-3′), and 5tr (5′-CTCCTGGGCTTCACGGAGAG-3′) confirmed the excision of exons 3 and 4 at the targeted sites. The WT allele yielded a product of 574 bp. The *Nudt7*^*+/−*^ mice were backcrossed onto a C57BL/6J background for six generations and then bred to generate *Nudt7*^*−/−*^ mice and *Nudt7*^*+/+*^ littermate controls.

Mice were housed at a room temperature of 22.2 ± 0.2 °C, room humidity of 40% ± 2%, and a 12-h light/12-h dark cycle, with the dark cycle starting at 6:00 PM. A WD (Research Diets D12079B, 41% kcal from fat, and 0.2% cholesterol,) or low-fat CD (Research Diets 98121701, 10% kcal from fat, 0% cholesterol) were fed to the *Nudt7*^*−/−*^ and WT mice starting at 6 weeks of age until they were sacrificed between 17 and 20 weeks of age. Age-matched male and female mice were used in all experiments. Mice were either harvested in the fed state at 7:00 AM or in the fasted state after being placed in cages with grids without food for 24 h. Food consumption was measured by individually housing mice in cages with grids, providing them with a pre-weighed amount of food and weighing the food remaining after 72 h. Feces collection was performed in conjunction with food monitoring studies. Blood glucose was measured either in the fed state or after fasting for 24 h. Body weight monitoring was initiated at 6 weeks of age, with repeated measurements taken every two weeks. Tissue harvest was initiated by administration of isoflurane, followed by blood collection by cardiac puncture and subsequent removal of the liver, gallbladder, and small intestine. Tissue samples were quickly weighed before flash-freezing in liquid nitrogen, then stored at −80 °C until analysis. Blood samples were allowed to clot, before being centrifuged at 10,000*g* for 10 min to isolate serum. All studies were approved by the Institutional Animal Care and Use Committees of West Virginia University.

### Targeted and untargeted metabolomics

For the analysis of the hepatic acyl-CoA pool composition, CoA species were extracted and analyzed as previously described (49). The bile acid pool composition of liver, gallbladder, and small intestine was analyzed as described by Zhang, Y. *et al* (110). Briefly, flash-frozen liver or small intestine samples (∼120 mg) were homogenized in 600 μl of water. For the small intestine, 60 μl of this homogenate were transferred to another tube to be used for the extraction. A deuterated bile acid internal standard mix was added to each homogenate (20.8 μl for liver samples, 58.3 μl for small intestine samples) such that the final concentrations of internal standards in the resuspended samples were 8 μg/ml of ^2^H_5_-β-MCA, 2 μg/ml of ^2^H_4_-CDCA, 4 μg/ml ^2^H_4_-TCDCA, 0.5 μg/ml of ^2^H_4_-ursodeoxycholic acid (UDCA), and 1 μg/ml ^2^H_4_-TUDCA. Alkaline acetonitrile (3 ml) was added to the homogenates, and the samples were shaken at 300 rpm at room temperature for 1 h. Following centrifugation at 1600*g* for 10 min, the supernatants were removed, while the pellets were resuspended in 2 ml of ethanol and sonicated using a probe sonicator set on pulse, the output control set at 3, and the duty cycle set at 50%. The sonicated pellets were spun down again, and the supernatants pooled with the previous fractions to be dried under nitrogen flow. Dry extracts were stored at −80 °C until analysis. Gallbladder samples were prepared by homogenization in 500 μl of 75% ethanol, followed by dilution (1:50) in 50% methanol to a volume of 100 μl, and by the addition of 8.3 μl of deuterated standard mix. The LC/MS/MS analysis was conducted in the Metabolome Analysis Facility at West Virginia University on an AB Sciex QTrap 5500 mass spectrometer connected to an AB Sciex Exion UPLC and controlled by Analyst Version 1.6.3 software. Frozen extracts were resuspended in 50% methanol (250 μl for liver samples, 700 μl for small intestine samples) and fractionated at 0.4 ml/min onto an Acquity UPLC BEH C18 column (2.1 × 100 mm, 1.7 μm particle size, Waters) kept at 45 °C and equilibrated with 92% buffer A (10 mM ammonium acetate in 20% acetonitrile, pH = 6.8) and 8% buffer B (10 mM ammonium acetate in 80% acetonitrile, pH = 6.8). Following injection of the samples (7.5 μl for liver and gallbladder samples, 3 μl for small intestine samples), the concentration of buffer B was maintained at 8% for an additional 3 min, then increased to 14% in 8 min, 25% in 3 min, 50% in 5 min, 60% in 2 min, and 80% in 1 min. The concentration of buffer B was maintained at 80% for 3 min before decreasing to the initial 8% in 3 min and allowing the column to equilibrate for 2 min. Bile acids were detected in negative mode using the following parameters: ion spray voltage of -3.5 kV, temperature of 550 °C, ion source gas 1 and 2 both set at 60, collision gas set at medium, and curtain gas set at 40. The collision cell exit potential was set to -13 V for all species. The entrance potential (EP) was set to -10 V for all species except HDCA (EP = −4 V) and ^2^H_4_-UDCA (EP = −8 V). The collision energy was set at −65 V for TCDCA and TMCA; −61 V for ^2^H_4_-TUDCA; −60V for TCA, TUDCA, THDCA, and ^2^H_4_-TCDCA; −55 V for TDCA and TLCA; −45 V for CA; −40 V for THCA, DHCA, CDCA, LCA; −35 V for UDCA and ^2^H_4_-UDCA; −30 V for α-MCA, β-MCA, DCA, HDCA, ^2^H_4_-CDCA, and ^2^H_5_-β-MCA. The retention times and Q1, Q3 mass transitions can be found in Table S1. Calibration curves for each bile acid were obtained at the beginning and end of each analysis and averaged. Peak analysis was conducted using MultiQuant 3.0.2. Peak areas corresponding to β-MCA and α-MCA were normalized to ^2^H_5_-β-MCA or ^2^H_4_-CDCA; peak areas corresponding to UDCA, HDCA, and CA were normalized to ^2^H_4_-UDCA; peak areas corresponding to CDCA, DCA, LCA, and THCA were normalized to ^2^H_4_-CDCA; peak areas corresponding to TMCA, TUDCA, THDCA, and TCA were normalized to ^2^H_4_-TUDCA; peak areas corresponding to TCDCA, TDCA, and TLCA were normalized to ^2^H_4_-TCDCA. Corrected peak areas were further normalized to the tissue weight and converted to absolute units using the calibration curves. The bile acid pool size of each organ was calculated by multiplying the concentration of each bile acid by the total organ weight, followed by the addition of all the products. The liver, gallbladder, and small intestine bile acid pools where then summed to obtain the total bile pool per mouse. To determine the total amount of CA derivatives, CA, TCA, DCA, and TDCA were combined across organs. All other bile acids were combined to determine the total amount of CDCA derivatives. The hydrophobicity index of the liver, gallbladder, and intestine bile acid pools was calculated from the molar fractions of the taurinated bile acid, as described by Heuman D. M (111). Serum levels of C4 were determined as described by Steiner C. *et al* (112), with minor modifications, in the Metabolome Analysis Facility at West Virginia University on an AB Sciex QTrap 5500 mass spectrometer connected to an AB Sciex Exion UPLC and controlled by Analyst Version 1.6.3 software. Briefly, 70 μl of serum were mixed with 416 μl of water, 3.6 μl of methanol, and 35 μl of a 0.5 μM solution of C4-d7 internal standard in methanol (Toronto Research Chemicals), followed by the addition of 42 μl of 1 M HCl. Solid phase extraction was performed using HF Bond Elut C18 columns (200 mg, Agilent technologies) and eluting the samples with 3 ml of methanol. The samples were dried under nitrogen flow and resuspended in 80 μl of methanol, before adding 10 μl of a 10 mM solution of ammonium acetate. The resuspended samples were incubated at room temperature for 10 min and then spun down at 20,000*g* for 10 min at 10 °C. A 65 μl aliquot of each sample was transferred to vials, and 10 μl was injected onto a Accucore RP-MS column (4.6 × 100 mm, 2.6 μm particle size, Thermo Fisher Scientific) kept at 25 °C and equilibrated in 5% buffer A (10 mM ammonium acetate, pH 6.5) and 95% of buffer B (methanol). This buffer composition was maintained for 12 min, with a flow rate of 0.4 ml/min. C4 and C4-d7 were eluted at 6.2 min and detected in positive mode, monitoring the transitions 401.3 to 383.3 *m/z* and 408.3 to 390.3 *m/z*, respectively. The following settings were used: ion spray voltage of 3.5 kV, temperature of 550 °C, ion source gas 1 and 2 both set at 60, collision gas set at medium, and curtain gas set at 40. The collision cell exit potential was set to 15 V, the EP was set to 13 V, and the collision energy was set to 30 V. A C4 calibration curve was obtained in the 6.25 to 400 nM range and used to quantitate the amount of C4 present in the serum samples. Global metabolomics profiling and related statistical and pathway enrichment analyses were conducted by Metabolon, Inc. Peak intensities normalized to tissue weights and significantly changed metabolites are reported in Table S2. Principal component analysis were conducted using MetaboAnalyst 5.0 (https://www.metaboanalyst.ca/) (113).

### Western blotting

Liver, heart, brown adipose tissue, and white adipose tissue samples (20–50 mg) were homogenized in 200 μl of radioimmunoprecipitation assay buffer supplemented with 1 × protease inhibitor cocktail. Small intestine and colon samples were homogenized in 200 μl of radioimmunoprecipitation assay buffer supplemented with 5 mM EDTA, 1 mM PMSF, and 5 × protease inhibitor cocktail. Homogenates were centrifuged at 10,000*g* for 10 min at 4 °C. Proteins (50–100 μg) were fractionated on 4 to 12% bis–Tris polyacrylamide gels, transferred onto PVDF membranes, and visualized by Ponceau S staining. The GAPDH antibody was used at a 1:3000 dilution and incubated at 4 °C overnight. The catalase and NUDT7 antibodies were used at a 1:500 and 1:6000 dilution, respectively, incubating for 1 h at room temperature. Horseradish peroxidase–conjugated goat anti-rabbit IgG was used as the secondary antibody at a 1:22,500 dilution, incubating for 1 h at room temperature. Antibody signals were detected by chemiluminescence on a G:BOX Chemi XX9 imaging system (Syngene). For quantification, the antibody signal in each sample was normalized to the total protein loaded, as determined by the intensity of the Ponceau S stain, and expressed relative to male mice fed the CD. Densitometric analysis was conducted using ImageJ (https://imagej.nih.gov/ij/).

### Histology and lipid analysis

Liver samples were harvested from mice in the fed state at 7:00 AM and immediately fixed in 10% neutral buffered formalin for 1 week at 4 °C. Fixed samples were embedded in paraffin blocks, cut into 5 μm sections, and stained with H&E by the WVU Histopathology Core Facility. The degree of macrovesicular steatosis was semiquantitatively scored based on the percentage of hepatocytes showing macrovesicular steatosis on a 0 to 4 scale as follows: 0 = 0%; 1 = 1 to 25%; 2 = 26 to 50%; 3 = 51 to 75%; 4 = 76 to 100%. Serum triacylglycerol and total cholesterol were measured using Stanbio kits (EKF Diagnostic USA), as per the manufacturer’s instructions. Liver triacylglycerol and total cholesterol were measured as previously described (49). To measure total fecal bile acids, feces (100 mg) were homogenized in 600 μl of water and bile acids extracted with alkaline acetonitrile as described above, but omitting the addition of deuterated internal standard mix. These extracts were resuspended in a mixture of 75% ethanol:PBS (1:4, v/v, 250 μl) and analyzed using the Mouse Total Bile Acid Assay Kit (Crystal Chem Inc.), as per manufacturer’s instruction. The content of triacylglycerol and free cholesterol in feces was measured, in duplicate, by modifying the protocol described by Argmann C. A. *et al* (114). Briefly, fecal samples were weighed (200–400 mg) and transferred to glass tubes with screw caps. A mixture of chloroform:methanol (2:1, v/v, 2 ml) was added to the feces, and samples were vortexed, transferred to a shaking water bath, and continuously shaken for 30 min at 60 °C. The lipid extract was filtered through Whatman grade 1 filter paper into glass test tubes, diluted to 4 ml with chloroform:methanol (2:1, v/v), and 1 ml of water was added. Samples were vortexed and centrifuged at room temperature for 10 min at 1600*g* to allow for phase separation. The lower phase was quantitatively removed, dried under nitrogen flow, and stored at −20 °C until analyzed. Samples were then resuspended in chloroform:methanol (2:1, v/v, 2 ml) and the lipids fractionated on HPTLC silica plates developed in hexane:ether:acetic acid (80:20:1, v/v/v). Resolved lipid bands and serial dilutions of tripalmitin and cholesterol standards were visualized by spraying with a 0.002% primulin solution followed by measurement of fluorescence using a G:BOX Chemi XX9 imaging system (Syngene).

### CoA-degrading and BSH activity assays

To monitor the production of 3′,5′-ADP in liver extracts, flash-frozen tissue (∼50 mg) was homogenized in plastic 1.5 ml tubes with a plastic homogenizer in 500 μl of 0.5% Triton X-100 in 20 mM Tris–HCl, pH 8. The homogenate was incubated on ice for 5 min and centrifuged at 20,000*g* for 15 min. The supernatant was then applied onto a Bio-Rad Econo-Pac 10DG desalting column equilibrated in 0.5% Triton X-100 in 20 mM Tris–HCl, pH 8, followed by the addition of 4 ml of the same buffer, and collection of the eluate in 0.5 ml fractions. The protein-containing fractions were then pooled and used for the assay. The standard assay contained 200 μg of desalted homogenate protein, 100 mM Tris–HCl, pH 8, 0.1 mg/ml γ-globulin, 2 mM MnCl_2_, and 250 μM of free CoA, in a total volume of 200 μl. The reactions were incubated at 37 °C for 10 min and stopped by the addition of perchloric acid, as previously described (24). Following neutralization with potassium carbonate, the reaction mixtures were diluted 4-fold with water and filtered through Spin-X 0.45 μm cellulose acetate centrifugal filters before being analyzed by HPLC (24).

Measurement of the BSH activity in the feces was performed as described elsewhere (115, 116). Briefly, mice were individually housed on wire grids for 24 h, at which point the feces were collected and stored at −20 °C. Fecal samples (∼75 mg each) were homogenized with a Dounce homogenizer in 1.5 ml of PBS supplemented with 20 mM β-mercaptoethanol and spun at 500*g* for 10 min to remove particulate. The supernatant was transferred to a clean tube and 100 μl were incubated at 37 °C for 2 h in a reaction mixture containing 4.1 mM sodium acetate, 20 mM β-mercaptoethanol, 0.41 mM EDTA, 0.91 mM sodium taurocholate, and 9.1 mg/ml of feces, in a total volume of 500 μl. The reactions were stopped by the addition of an equal volume of 20% trichloroacetic acid and 100 μl mixed with 1 ml of ninhydrin reagent (1:1 mixture of 0.16% stannous chloride in 200 mM sodium citrate and 4% ninhydrin in 2-methoxyethanol). Following a 30 min incubation at 95 °C, the absorbance at 600 nm was measured. For each fecal extract, background taurine was estimated in reaction mixtures where sodium taurocholate was replaced by water and subtracted. Taurine calibration curves were obtained in the 0 to 100 nmoles range and used to calculate the nmoles of taurine released per mg of feces.

### Measurement of total liver CoA levels and the rate of fatty acid oxidation

Total hepatic CoA levels were measured as previously described (117). To measure the rate of fatty acid β-oxidation, primary hepatocytes were isolated from ad libitum–fed mice or mice fasted for 24 h, between 7:00 and 8:00 AM, as previously described (49, 56). Female mice were fasted for 14 to 16 h before hepatocyte isolation, as a longer fast consistently decreased the yield of viable hepatocytes, regardless of the genotype. Hepatocytes isolated from ad libitum–fed mice were assayed for the rate of fatty acid β-oxidation in medium supplemented with 1 g/l of glucose. No additional glucose was added to the medium used to assay hepatocytes isolated from fasted mice (49, 56).

### RT-qPCR and RNA-seq

For RT-qPCR, RNA was isolated from flash frozen tissue using the TRI Reagent, as per manufacturer’s instructions. Following the removal of genomic DNA with Turbo DNA-free kit (Thermo Fisher Scientific), mRNA levels were quantified by RT-qPCR using the Quantitect SYBR Green RT-PCR kit (Qiagen). The relative abundance of each gene was calculated from triplicate measurements using the C_T_ method, and the amount (2^-ΔCT^) reported relative to the average of ribosomal protein L22 (*Rpl22*) and β2-microglobulin (*B2m*) transcript levels. The primer sequences used for mouse *Nudt7*, *Nudt8*, *Nudt19*, *Rpl22*, and *B2m* were previously published (25, 118). Primers 5′-GCCATCAAGGACGTCAGCA-3′ and 5′-CTTCCTCCGAGTAGCGAATCAG-3′ were used as forward and reverse primers, respectively, for *Fgf15*; primers 5′-TCTGCAGGTCGTCCGACTATTC-3′ and 5′-AGGCAGTGGCTGTGAGATGC-3′ were used as forward and reverse primers, respectively, for *Shp* (*Nr0b2*); primers 5′-TGGGGATGTTGGCTGAATGT-3′ and 5′-TGCCGTGAGTTCCGTTTTCT-3′ were used as forward and reverse primers, respectively, for *Fxr* (*Nr1h4*).

For RNA-Seq experiments, RNA was isolated from mouse liver as described above and sequenced by the West Virginia University Genomics Core Facility. Total RNA was used for library construction using the KAPA mRNA HyperPrep Kit (Roche) and run on the HiSeq 2500 (Illumina) in 50 bp paired end reads. Fastq files were aligned and mapped using Salmon (v1.3.0) (119). Briefly, a decoy-aware index was prepared using the *Mus musculus* primary genome assembly (Ensembl v101). Salmon was run using quantification mode, with no other parameter alterations. Tximport (v1.16.1) (120) and tximeta (v1.6.3) (121) were implemented to import transcript-level abundance and estimated counts, apply gene level information, and collate the quantification data. Differential expression analysis was performed using DESeq2 (v1.28.1) (122), and data were filtered for low abundance transcripts using "keep <- rowSums(counts(dds) ≥ 10) ≥ x", with a minimum read count of 10 for at least half of the samples within the dataset being analyzed. The DESeq function of DESeq2 utilizes three primary steps when evaluating significance between groups, including 1) sample- and gene-dependent normalization factors, 2) gene-specific dispersion, and 3) negative binomial generalized linear model fitting. This function provides estimates of dispersion and logarithmic fold changes that incorporate data-driven prior distributions. Pathway analyses was performed using Kyoto Encyclopedia of Genes and Genomes (KEGG v1.1.1) (123) and were visualized using pathfindR (v1.6.1) (124). The bioinformatic pipeline was executed in R (v4.0.3) and the code is provided at https://github.com/qahathaway/Nudt7-Western-Diet. The sequencing files and differential expression analysis are included within the GEO database, accession number GSE207914.

### Statistical analysis

Statistical significance between two groups was calculated using an unpaired two-tailed Student’s *t* test. WT and KO mice under different nutritional or dietary conditions were compared using a two-way ANOVA, followed by a Tukey’s multiple comparisons test. For the untargeted metabolomics analysis, a three-way ANOVA with genotype, gender, and diet as the main effect terms was used. For pathway enrichment analysis, a right-tailed Fisher’s exact test (RNA-Seq data) or cumulative hypergeometric distribution test (untargeted metabolomics) were used. A *p* value < 0.05 was considered statistically significant. For RNA-Seq analyses, the false-discovery rate was set to 0.05 and all significance was determined through an adjusted *p* value (*padj*) < 0.05. Bar graphs show the mean ± the SD, with individual biological replicates shown as circles.

## Data availability

The RNA-seq files and differential expression analysis are included within the GEO database, accession number GSE207914. All other data described are contained within the article.

## Supporting information

This article contains supporting information.

## Conflict of interest

The authors declare that they have no conflicts of interest with the contents of this article.
