# Supplementary material for: NUDT7 regulates total hepatic CoA levels and the composition of the intestinal bile acid pool in male mice fed a Western diet
Source: J Biol Chem. 2022 Nov 24;299(1):102745. doi: 10.1016/j.jbc.2022.102745 (PMC9792899; doi:10.1016/j.jbc.2022.102745)
Supplement: Supplemental Figures [file mmc4.pdf]

NUDT7 regulates total hepatic CoA levels and the composition of the intestinal bile acid pool in male mice fed a Western diet

Schuyler D. Vickers, Stephanie A. Shumar, Dominique C. Saporito, Amina Kunovac, Quincy A. Hathaway, Breeanna Mintmier, Judy A. King, Rachel D. King, Vazhaikkurichi M. Rajendran, Aniello M. Infante, John M. Hollander, Roberta Leonardi

File content:

Supplemental Figure S1: Free CoA, acetyl-CoA, and long-chain acyl-CoA composition

Supplemental Figure S2: Hepatic NUDT7 expression and gene expression changes in response to WD feeding in male and female mice

Supplemental Figure S3: Bile acid composition and pool size of liver and gallbladder

Supplemental Figure S4: Effect of Nudt7 deletion on bile acid signaling

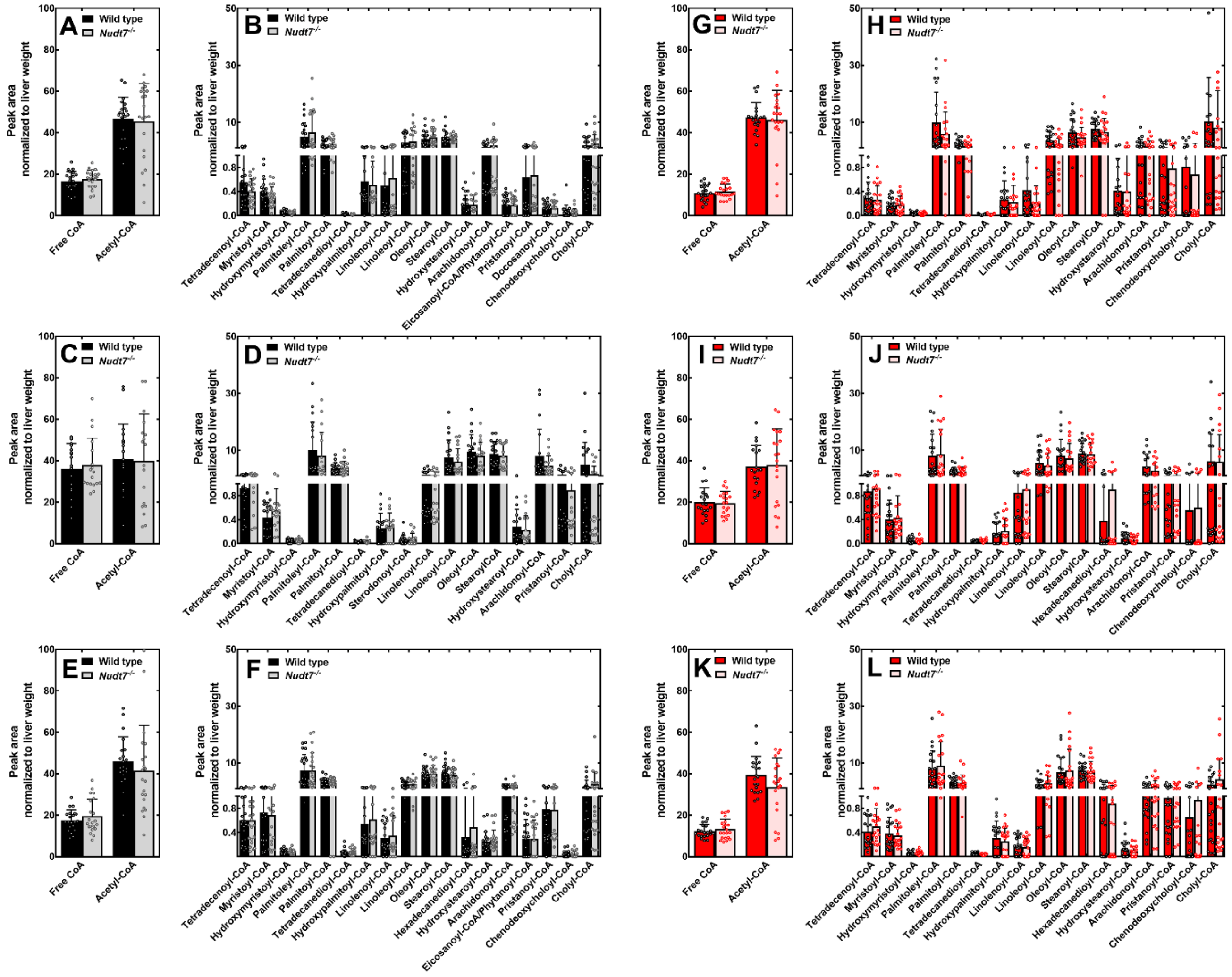

Supplemental Figure S1: Free CoA, acetyl-CoA, and long-chain acyl-CoA composition. (A and B) Levels of free CoA, acetyl-CoA (A), and long-chain acyl-CoAs (B) in the livers of *ad libitum* fed male mice fed the CD. (C and D) Levels of free CoA, acetyl-CoA (C), and long-chain acyl-CoAs (D) in the livers of male mice fed the CD and fasted for 24 h. (E and F) Levels of free CoA, acetyl-CoA (E), and long-chain acyl-CoAs (F) in the livers of *ad libitum* fed male mice fed the WD. (G and H) Levels of free CoA, acetyl-CoA (G), and long-chain acyl-CoAs (H) in the livers of *ad libitum* fed female mice fed the CD. (I and J) Levels of free CoA, acetyl-CoA (I), and long-chain acyl-CoAs (J) in the livers of female mice fed the CD and fasted for 24 h. (K and L) Levels of free CoA, acetyl-CoA (K), and long-chain acyl-CoAs (L) in the livers of *ad libitum* fed female mice fed the WD. Data are shown as the mean (bar) of measurements conducted on individual mice (circles) ± SD. Student's *t* test.

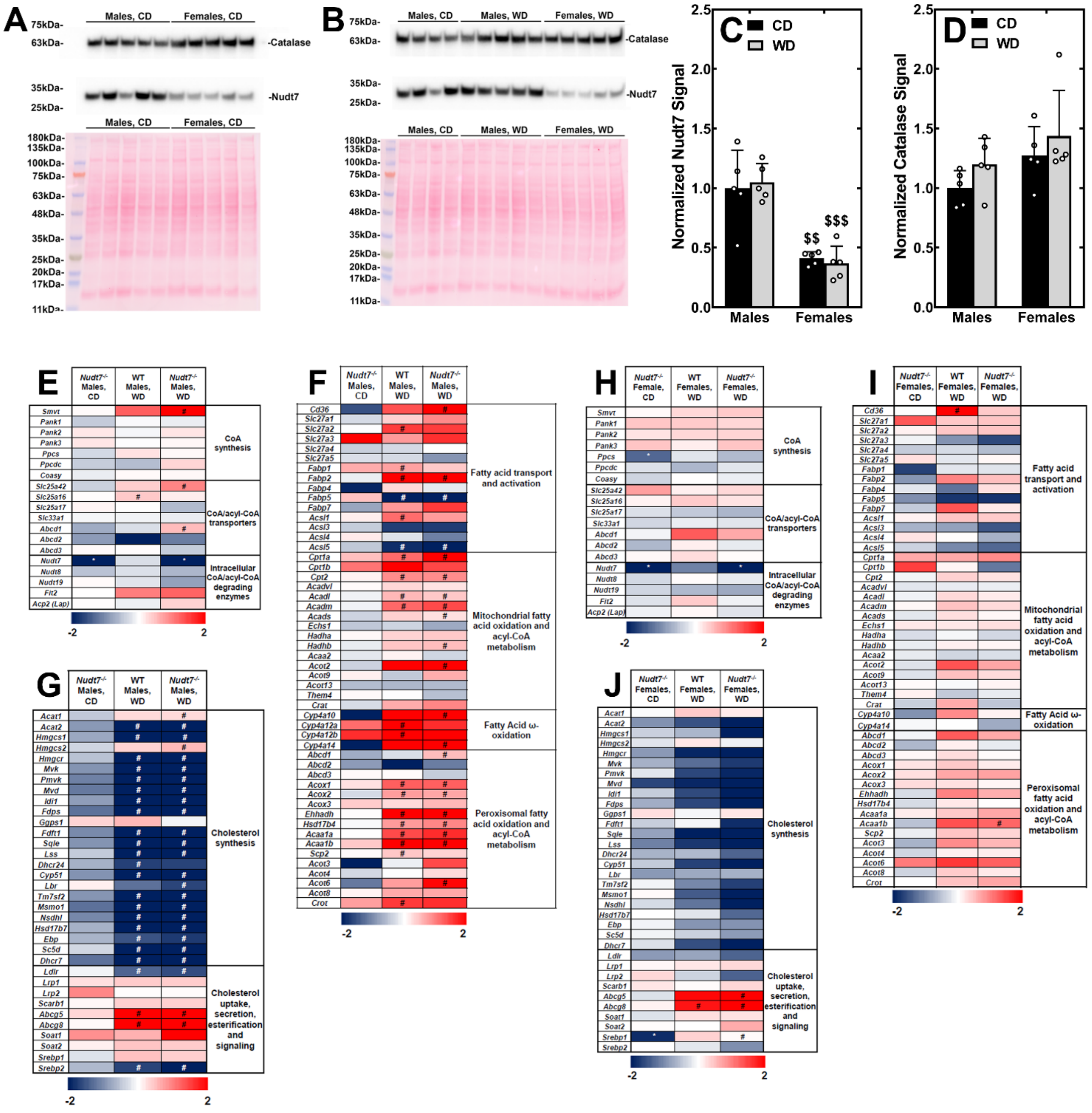

Supplemental Figure S2: Hepatic NUDT7 expression and gene expression changes in response to WD feeding in male and female mice. (A-B) Western blot analysis of livers from wild type mice on CD (A) or WD (B). Quantification of NUDT7 (C) and catalase (D) protein levels. Western blot signals were normalized to total protein, and expressed relative to WT males fed the CD. Data are shown as the mean (bar) of measurements conducted on individual mice (circles) ± SD. Two-way ANOVA, $$ p<0.01, $$$ p<0.001 between males and females fed the same diet. (E and H) Changes in the expression of genes involved in CoA synthesis, degradation and organellar import in the liver of wild type or *Nudt7*$^{-/-}$ males (E) and females (H) fed the CD or WD, relative to gender-matched control mice fed the CD. See also **supplemental Table**

S3. (F and I) Changes in the expression of genes involved in fatty acid transport and oxidation in the liver of wild type or *Nudt7*$^{-/-}$ males (F) and females (I) fed the CD or WD, relative to gender-matched control mice fed the CD. See also **supplemental Table S3**. (G and J) Changes in the expression of genes involved in cholesterol synthesis, uptake, secretion, and select metabolism in the liver of wild type or *Nudt7*$^{-/-}$ males (G) and females (J) fed the CD or WD, relative to gender-matched control mice fed the CD. See also **supplemental Table S3**. False discovery rate, # $p<0.05$ comparing mice of the same genotype fed the WD vs the CD; *, $p<0.05$ comparing mice of different genotypes fed the same diet.

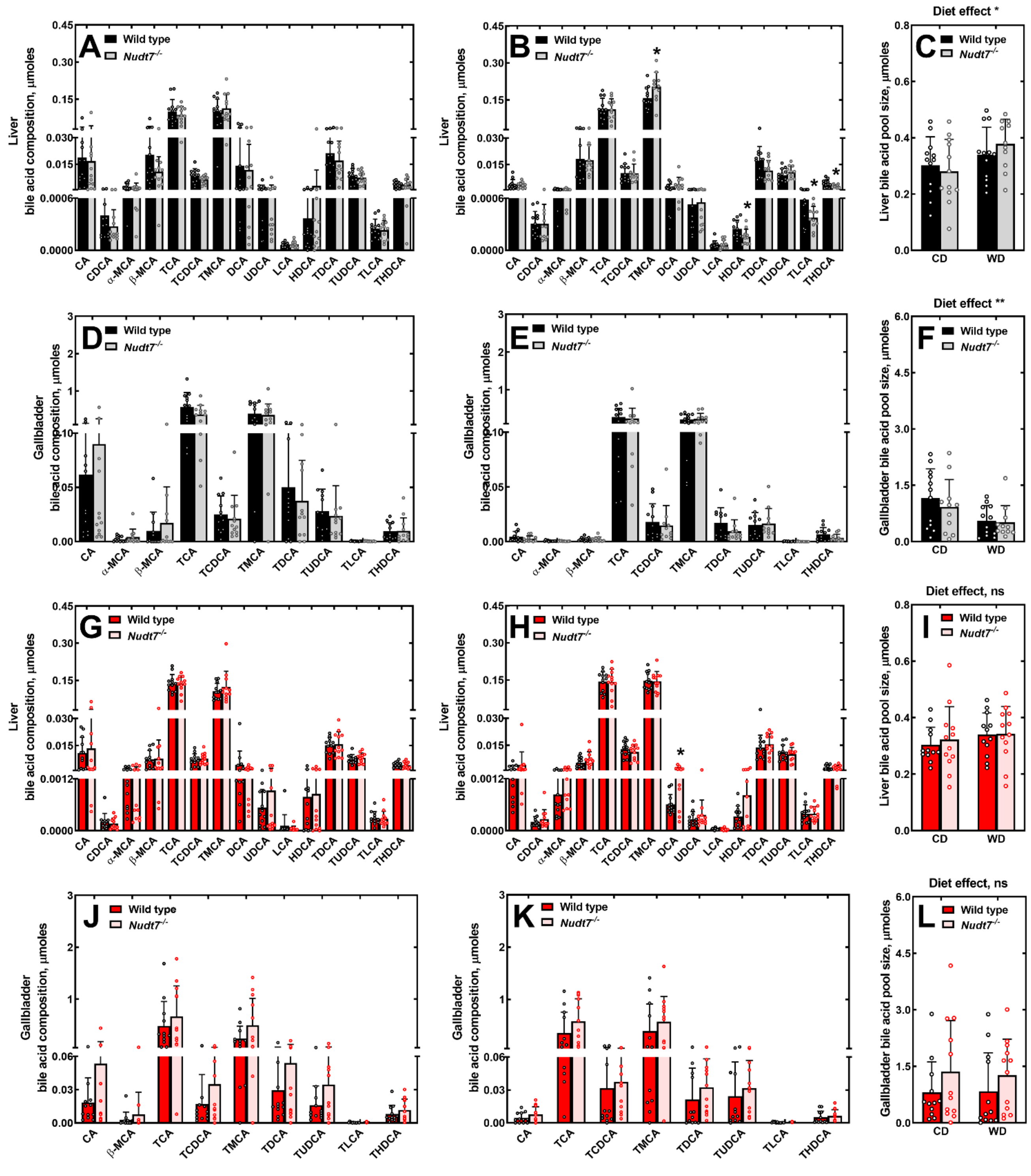

Supplemental Figure S3: Bile acid composition and pool size of liver and gallbladder. (A and B) Bile acid compositions of the livers of males fed the CD (A) and the WD (B). Student's t test, * *p*< 0.05. (C) Liver bile acid pool size in males fed the CD and the WD. Two-way ANOVA, * *p*<0.05 for diet effect. (D and E) Bile acid compositions of the gall bladders of males fed the CD (D) and the WD (E). Student's *t* test. (F) Gall bladder bile acid pool size in males fed the CD

and the WD. Two-way ANOVA, ** $p<0.01$ for diet effect. (G and H) Bile acid compositions of the livers of females fed the CD (G) and the WD (H). Student's *t* test, * $p<0.05$. (I) Liver bile acid pool size in females fed the CD and the WD. Two-way ANOVA, ns, not significant. (J and K) Bile acid compositions of the gall bladders of females fed the CD (J) and the WD (K). Student's *t* test. (L) Gall bladder bile acid pool size in females fed the CD and the WD. Two-way ANOVA, ns, not significant. Data are shown as the mean (bar) of measurements conducted on individual mice (circles) ± SD.

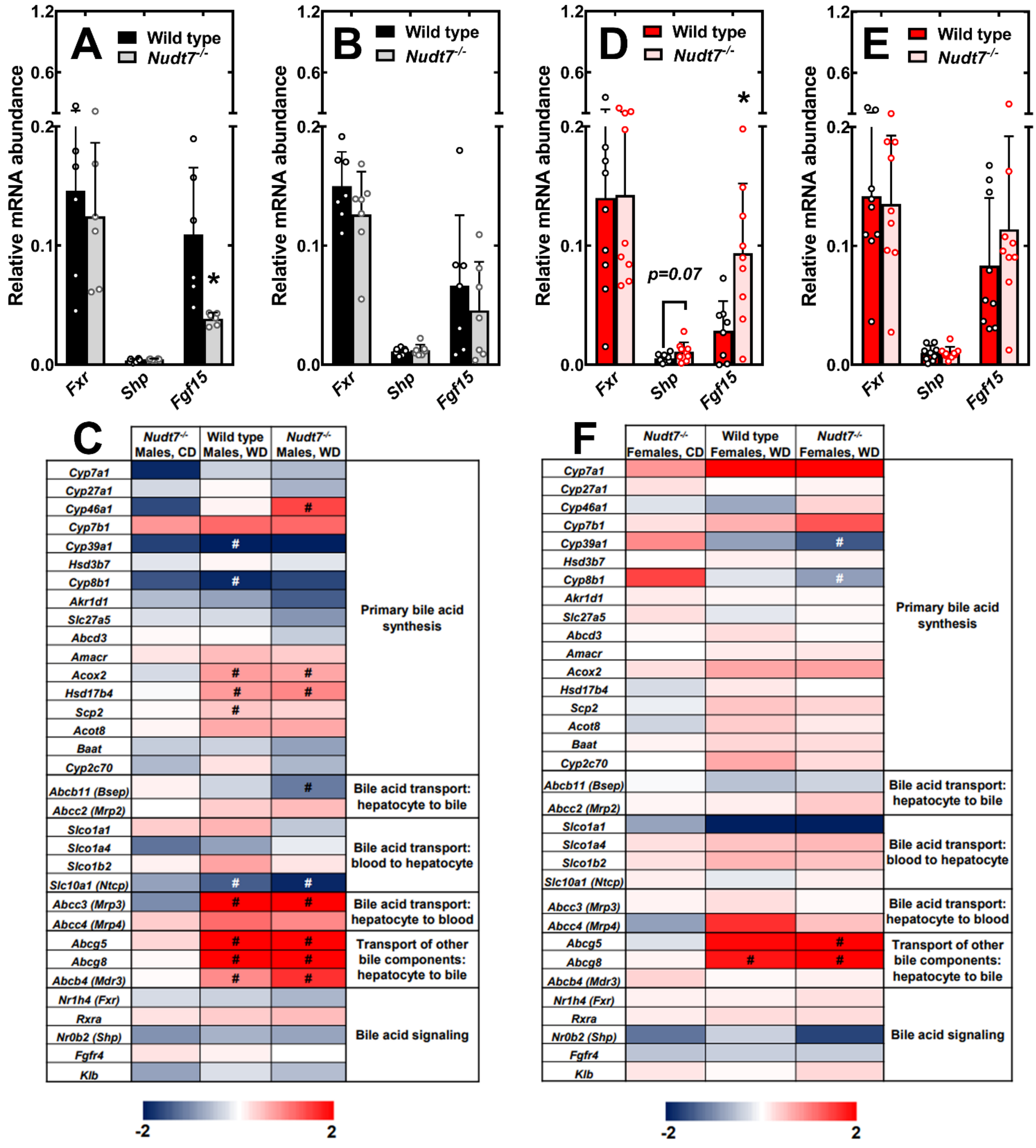

Supplemental Figure S4: Effect of Nudt7 deletion on bile acid signaling. (A and B) RT-qPCR analysis of *Fxr*, *Shp* and *Fgf15* transcripts in the small intestine of males fed the CD (A), or the WD (B). Data are shown as the mean (bar) of measurements conducted on individual mice (circles) ± SD. Student's *t* test, * $p<0.05$. (C) Changes in the expression of genes involved bile acid synthesis, transport and signaling in the liver of wild type or *Nudt7*$^{-/-}$ males fed the CD or WD, relative to wild type males fed the CD. See also **supplemental Table S3**. False discovery rate, # $p<0.05$ comparing mice of

the same genotype fed the WD vs the CD. (D and E) RT-qPCR analysis of *Fxr*, *Shp* and *Fgf15* transcripts in the small intestine of females fed the CD (D), or the WD (E). Data are shown as the mean (bar) of measurements conducted on individual mice (circles) ± SD. Student's *t* test, * $p<0.05$. (F) Changes in the expression of genes involved bile acid synthesis, transport and signaling in the liver of wild type or $Nudt7^{-/-}$ females fed the CD or WD, relative to wild type females fed the CD. See also **supplemental Table S3**. False discovery rate, # $p<0.05$ comparing mice of the same genotype fed the WD vs the CD.
